# Clustering Diagnoses From 58 Million Patient Visits in Finland Between 2015 and 2018

**DOI:** 10.2196/35422

**Published:** 2022-05-04

**Authors:** Pasi Fränti, Sami Sieranoja, Katja Wikström, Tiina Laatikainen

**Affiliations:** 1 Machine Learning Group School of Computing University of Eastern Finland Joensuu Finland; 2 Institute of Public Health and Clinical Nutrition University of Eastern Finland Kuopio Finland; 3 The Department of Public Health and Welfare Finnish Institute for Health and Welfare Helsinki Finland

**Keywords:** multimorbidity, cluster analysis, disease co-occurrence, multimorbidity network, health care data analysis, graph clustering, k-means, data analysis, cluster, machine learning, comorbidity, register, big data, Finland, Europe, health record

## Abstract

**Background:**

Multiple chronic diseases in patients are a major burden on the health service system. Currently, diseases are mostly treated separately without paying sufficient attention to their relationships, which results in the fragmentation of the care process. The better integration of services can lead to the more effective organization of the overall health care system.

**Objective:**

This study aimed to analyze the connections between diseases based on their co-occurrences to support decision-makers in better organizing health care services.

**Methods:**

We performed a cluster analysis of diagnoses by using data from the Finnish Health Care Registers for primary and specialized health care visits and inpatient care. The target population of this study comprised those 3.8 million individuals (3,835,531/5,487,308, 69.90% of the whole population) aged ≥18 years who used health care services from the years 2015 to 2018. They had a total of 58 million visits. Clustering was performed based on the co-occurrence of diagnoses. The more the same pair of diagnoses appeared in the records of the same patients, the more the diagnoses correlated with each other. On the basis of the co-occurrences, we calculated the relative risk of each pair of diagnoses and clustered the data by using a graph-based clustering algorithm called the M-algorithm—a variant of k-means.

**Results:**

The results revealed multimorbidity clusters, of which some were expected (eg, one representing hypertensive and cardiovascular diseases). Other clusters were more unexpected, such as the cluster containing lower respiratory tract diseases and systemic connective tissue disorders. The annual cost of all clusters was €10.0 billion, and the costliest cluster was cardiovascular and metabolic problems, costing €2.3 billion.

**Conclusions:**

The method and the achieved results provide new insights into identifying key multimorbidity groups, especially those resulting in burden and costs in health care services.

## Introduction

### Multimorbidity

Multiple chronic diseases in patients are a major burden to the health service system in terms of both service use and costs [1]. In many service systems, diseases are mostly treated separately without paying sufficient attention to their relationships, which results in the fragmentation of the care process. Better integration of services can lead to a more effective organization of the overall health care system. To support this, we analyzed the connections between diseases based on their co-occurrence and performed a clustering analysis to identify multimorbidity patterns.

Multimorbidity is often defined as the coexistence of ≥2 chronic conditions within a patient [2,3]; however, the number of medical conditions included in this definition ranges widely [4]. Systematic reviews have shown that multimorbidity reduces self-rated health, quality of life, and functional ability and increases the risk of premature death, hospitalization, and use of health services, causing a substantial economic burden for societies and health care systems [5]. Wang et al [6] reported that multimorbidity cases, defined as patients with ≥2 chronic conditions, have 2 to 16 times higher costs than nonmultimorbidity cases. Brettschneider et al [7] analyzed the impact of 45 conditions on health-related quality of life. The authors measured multimorbidity using a weighted count score and assessed its association with decreases in the health-related quality of life. The strongest impact was observed in Parkinson disease, depression, and obesity.

An active research area is the measurement of the severity of multimorbidity. Stirland et al [8] reviewed 35 multimorbidity measures. Most measures (25 of 35) in their review were based on simple (weighted or unweighted) counts of diseases; some measures (4 of 35) used drug counts, and some (5 of 35) were based on expert-generated grouping of diagnoses, mainly based on frequencies. Such measures have been used to assess mortality, health care use, cost, and quality of life.

### Diagnosis Groups

The number of possible multimorbidities is too large for human analysts to examine them individually. In the case of only 205 diagnoses, there are 20,910 different pairs of diagnoses. It is easier to analyze their connections by first dividing the diagnoses into smaller groups that contain related diagnoses and then examining only the connections between diagnoses within each group. This effectively removes less relevant multimorbidities from the data and allows us to show the connections in small groups that are easy to analyze.

Diagnosis groups can also predict future costs for a patient. Farley [9] discovered that simply counting the number of diagnosis clusters to which a patient belongs is a good predictor of high costs in the future. When combined with other measures such as the number of prescriptions, it outperformed more complex comorbidity indices such as the Charlson, Elixhauser, and RxRisk-V indices [9].

Diagnosis groups were previously created manually by experts by joining diagnoses of clinical similarity. Travers et al [10] studied how well the 4 groupings covered emergency medicine. The authors discovered that the Agency for Healthcare Research and Quality grouping for inpatient care provides the best coverage (99%), whereas the National Center for Health Statistics vital statistics grouping covers only 88%. They also criticized that most clusters (76%) were small, and there were large clusters containing dissimilar conditions. Open questions include how to evaluate a cluster system and determine its clinical relevance. Travers et al [10] further argued that a good clustering system should collapse the individual International Classification of Diseases, Ninth Revision, Clinical Modification (ICD-9-CM) codes into clinically meaningful clusters.

The number of groups was also problematic. Schneeweiss et al [11] argued that 367 clusters are too many for comparative analysis, whereas 17 clusters are too broad for this purpose. The authors reduced the number of International Classification of Diseases (ICD) categories to 110 diagnosis clusters by cross-tabulation between the ICD-9-CM and International Classification of Health Problems in Primary Care–2 classifications, covering approximately 90% of all diagnoses of their records made by family physicians.

### Clustering to Detect Multimorbidity Patterns

An alternative to the manual grouping of diagnoses is the use of computer algorithms to create groups. A cluster is a group of objects that are similar to each other, whereas objects in different clusters are expected to be far from each other or at least less similar than those in the same cluster [12]. Clustering can be used to detect multimorbidity patterns by grouping either patients or diseases [13]. If we group the diagnoses, one diagnosis belongs to only one cluster, whereas a patient can belong to several clusters. If we group the patients, the reverse is true: one diagnosis can belong to several groups, but one patient can belong to only one cluster. This study focused on grouping diagnoses.

The data used in clustering can be either numerical values or text. Here, we follow the study by Hidalgo et al [14] and represent the diagnoses as nodes and their relationships as links in a network. We refer to this as the *multimorbidity network*. In this network, the weight of the links between 2 diagnoses measures how strongly they correlate in a patient record database.

Although clustering algorithms have been widely used elsewhere in health care, the existing literature lacks reliable, automatic, and computer-generated clusters. Estiri et al [15] used clustering to detect anomalies in health records by combining agglomerative clustering with a k-means algorithm. The idea was to detect small clusters and flag them as anomalies. The authors reported a significantly smaller number of false positive cases than simple anomaly detection based on the SD and Mahalanobis distance.

Huang et al [16] clustered patients into 5 clinically meaningful groups based on the similarity of their diagnoses and the geographical locations of the hospitals. Their motivation was to build machine learning models trained for each group separately to provide a better prediction of mortality and intensive care unit stay time.

Kalgotra et al [17] used co-occurrence statistics to build a *multimorbidity network* to study the disparity of gender. The statistics were extracted from the treatment data of >22.1 million patients. They created networks separately for men and women and compared the structures of the 2 networks. The networks of female patients had more connections with mental health.

Folino et al [18] clustered patients based on a multimorbidity network built using co-occurrence statistics. They used the k-means clustering algorithm with Jaccard distance. A representative of each cluster was chosen as the set of all diseases whose relative frequency in the cluster exceeded a user-defined threshold (eg, 0.8). Clustering was used to predict future diseases and was tested using the records of 1462 patients from a small town in South Italy.

In the study by Folino and Pizzuti [19], the same prediction system was revised using common neighbors in the network. Records of 2541 patients from 2000 to 2009 were used to build a network from ICD-9-CM codes. The resulting network contained 492 nodes and 21,676 connections. A total of 2 separate subnetworks were created. The first included only connections with *a relative risk* (RR) value of >20 (2330 connections), and the other included those with a Pearson correlation value of ≤0.06 (7242 connections). Future patient diseases were predicted by calculating the number of common neighbors shared by the 2 diseases.

Ding et al [20] extended the previous prediction model using ICD, 10th Revision (ICD-10) and demographic data. On the basis of data collected between 2007 and 2014 in an (unnamed) provincial capital in China, they reported that 71% of acute diseases and 82% of chronic diseases were predictable.

John et al [21] applied clustering to 1039 American Indians using data from an interview-based questionnaire. Cornell et al [20] used ICD-9 codes from data obtained from administrative databases of primary care clinics. Marengoni et al [22] used electronic medical records of the acute care wards of 38 internal medicine and geriatric wards in Italy in 2008.

Marengoni et al [22] calculated clusters of diseases to detect groups of patients at risk of in-hospital death. Their data comprised 1332 older people hospitalized in acute care wards. This small data set had 19 diagnoses, which were grouped into 8 clusters using a correlation matrix and average linkage agglomerative clustering. The results included 4 clusters comprising a disease and its possible consequences. For example, diabetes is clustered with cerebrovascular diseases and coronary heart diseases, thyroid dysfunction with anxiety, and chronic renal failure with anemia. The combination of chronic renal failure and anemia had the highest likelihood of in-hospital death, with an odds ratio of 6.1.

Most existing studies on clustering are based on hierarchical agglomerative methods using heuristic criteria, either *average* or *complete linkage* [13]. Wartelle et al [23] extended hierarchical agglomerative clustering by directly optimizing clustering using RR. By default, this is a more solid approach than any linkage criterion (single, average, or complete). They applied the method to data collected from the emergency department (ED) of Troyes Hospital in Eastern France during a 2-year period between 2017 and 2019. A network comprising 151 ICD-10 blocks was created using 114,391 hospital visits of 72,666 patients.

### Proposed Methodology

In this study, instead of agglomerative clustering, we applied a *k-means*–based algorithm. Previously, k-means clustering was used for clustering patients [24]. We applied the algorithm for clustering diseases using data comprising 45 million health care visits covering all public health service use (both primary and secondary care) of the population aged ≥18 years in the entire of Finland from 2015 to 2018. This data set is significantly larger than that used in any of the previous studies.

We constructed a multimorbidity network comprising diseases represented as blocks of the ICD-10 codes. Correlated diseases were in the network. The strength of the links between the diseases was measured using RR, which estimates how much higher the observed prevalence is in relation to the expected prevalence. Clustering was used to find multimorbidity patterns by dividing the network into subgroups with high RR values within. These groups can contain previously unknown multimorbidity patterns.

Similar to the study by Wartelle et al [23], our study was also based on RR. However, there were 2 main differences. First, the agglomerative clustering algorithm in the study by Wartelle et al [23] needs to access the original data after each merge to recalculate the RR values, which is very time consuming with large data. We constructed the network only once, without any need to access the original data after that. This approach scales better as the network is remarkably smaller than the original data (205 nodes vs 58 million patients). K-means itself may require multiple runs [25] to create accurate clustering; however, we avoided this by using a more robust derivation called the M-algorithm [26].

The second difference is that the results of [23] were obtained from emergency visits. Although the resulting clusters could be valid in this context, the generated clusters were different from those obtained from all general health care visits.

The main contributions of our paper can be summarized as follows:

- We use a k-means–based algorithm called M-algorithm, which has been shown to provide highly accurate clustering with controlled validation data sets and scaling up to large-scale data [26].
- We use inverse internal weight (IIW) in the network as a cost function as it has been shown to provide more balanced cluster sizes than other alternatives [26].
- We apply the algorithm to large-scale data comprising 58 million health care visits in all of Finland from 2015 to 2018.
- We make the data publicly available on the University of Eastern Finland website [27], including the multimorbidity network and the clusters.

These contributions directly support several of the goals described by Whitty and Watt [28]. These objectives include strengthening statistical methods to detect clusters, applying them to large data sets, and treating clusters of diseases more effectively. In this paper, we describe the content of the generated clusters and their relationships with nearby clusters. We report the most significant observations and their effects on both service use and costs in the health care system. The study follows the TRIPOD (Transparent Reporting of a multivariable prediction model for Individual Prognosis or Diagnosis) guidelines [29] for all relevant items except those related to prediction.

## Methods

### Overview

Graph clustering has been used in physics [30,31], engineering [32], image processing [33], and medical [34] and social sciences [35]. The technique has several names, including *network community detection* [36-42], *graph clustering* [43] or *graph partitioning* [33,44,45]. These methods can be directly applied to diseases by considering the co-occurrence matrix of diseases as a graph.

By grouping data into meaningful clusters and finding co-occurring diagnoses, it is possible to plan the treatment processes of multimorbid patients and the resources needed in service provision. It is known that diseases often cluster because of a common risk factor; however, only a small number of possible clusters and the connections between the clusters are well known [28].

### Data

A summary of the patient record database is presented in Table 1. The data were extracted from the National Administrative Care Register for Health Care, covering all inpatient and outpatient primary and specialized care between 2015 and 2018. Finnish health care registers include data on the patient’s age, gender, and the municipality of residence, as well as information concerning the service event, such as the type of contact (visit, phone call, or inpatient admission) and reason for the visit, treatment, and procedures. Reasons for visits were recorded using ICD-10 or International Classification of Primary Care, second edition codes.

**Table 1:** Summary of the patient database.

Data			Values
**Entire database**			
	**All patients, n (%)**		4,280,985 (100)
		Patients with ICD-10^a^ codes	3,987,382 (93.14)
	Time range		2015 to 2018
	**Total visits, n (%)**		311,721,962 (100)
		Visits with ICD-10 codes	69,306,854 (22.23)
	Number of diagnoses per visit, mean		1.6
	Total cost of all visits per year (€^b^)		9685 million
**Included in clustering**			
	Visits, n (%)		58,391,604 (18.73)
	Costs per year (€)		6596 million
	Cost of patient per year (€), mean (SD)		2538 (6478)
	Patients, n (%)		3,835,531 (89.59)
	Patients per year, mean (SD)		2,536,944 (37,494)
	**Gender, n (%)**		
		Women	2,062,110 (54)
		Men	1,773,419 (46)
	Age (years), median		54
	Patients aged >70 years, n (%)		943,717 (25)

^a^ICD-10: International Classification of Diseases, 10th Revision.

^b^A currency exchange rate of €1=US $1.09 is applicable.

The entire patient record database contains information on 4.3 million patients aged >18 years. For the cluster analysis, we only included patients with a medical diagnosis (excluding external cause diagnoses), which totaled 3.8 million. The full database included approximately 312 million contacts with health services. The visits were divided into 272,090,337 contacts with primary care services and 39,631,625 contacts with special care services. Primary care contacts included 142,874,297 home visits, 71,658,708 visits to a health center, 26,849,249 phone calls, and 30,708,083 other types of contacts.

For the clustering analysis, from all the visits (311,721,962), we included only those having ICD-10 diagnoses recorded (n=69,306,854 [22.23%]). We excluded all the symptom codes (R00-R99); external causes for injuries, diseases, and deaths (V01-Y92); and health factors and contacts to the service providers (Z00-ZZB), as they do not represent any disease themselves, as well as special diagnosis codes (U00-U99). After filtering these out, the remaining data included 18.73% (58,391,604/311,721,962) of visits.

The costs for each diagnosis were calculated using the computational standard cost [46,47] using patient grouping methods and standard unit costs calculated from national-level cost accounting projects. Hospitalizations and hospital outpatient visits were grouped using the Nordic Diagnosis-Related Groups grouper. The Nordic Diagnosis-Related Groups cost weights for hospitalizations and outpatient visits were based on individual-level cost accounting data from several hospitals and were used in the national price lists by the Finnish Institute for Health and Welfare [48]. The unit cost estimates for each type of primary care contact were obtained from the national standard price list for primary care encounters. The unit cost estimates for social care encounters and community care bed-days were derived from the national price list for the unit costs of health care services in Finland.

The total annual health service cost in Finland during the period 2015 to 2018 was €9685 million for a total of 311 million visits. A currency exchange rate of €1=US $1.09 is applicable. The cost estimation for the data used in the cluster analysis totals to €6596 million per year. The annual cost of each year had an increasing trend between 2015 and 2017 but decreased in 2018: €6579 million (2015), €6626 million (2016), €6723 million (2017), and €6455 million (2018). Some changes may have originated from changes in recording practices. In addition, patients who were hospitalized for longer periods (weeks or months) were not included in the 2018 data if they were not discharged by the end of 2018.

### Measuring RR

There are several possibilities for measuring the strength of the relationship between 2 diseases (Table 2). These include *φ correlation* (Pearson correlation) [14,34], *co-occurrence correlation* [49], *Jaccard coefficient* [50], *Yule Q* [21,22], *Salton cosine index* [17], and multiple variants of RR [18,19,26]. For a good review, refer to the study by Srinivasan et al [49].

**Table 2:** Ways of measuring disease connectivity.

Name	Formula^a^	References
Relative risk 1	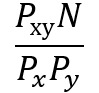	[14,51]
Relative risk 2	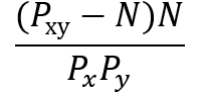	[18]
Relative risk 3	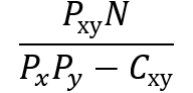	[52]
Co-occurrence correlation	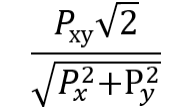	[49]
φ-correlation	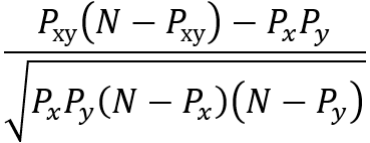	[14,18,34] (slight variation [52])

^a^*N*: number of patients; *P*_x_: number of patients with diagnosis *x* (prevalence); *P_xy_*: number of patients with both diagnosis *x* and *y* (prevalence); E[*xy*]: expected frequency of *xy*; *p*(*x*)=*P*_x_/*N*: probability of a random patient having a diagnosis x; *p*(*xy*)=*P*_xy_/*N*: probability of a random patient having both diagnosis *x* and *y.*

Several authors [17,23,49] have noted that the existing measures contain biases. For example, RR overemphasizes the connection between infrequent diseases. The Pearson correlation underestimates the relationship between common and infrequent diseases. Owing to these problems, Srinivasan et al [49] ended up proposing their own method, called *co-occurrence correlation*.

We used RR (variant 1 in Table 2) as this measure has been widely used in the literature, and its values are clear to understand. It has been used previously by several authors [14,18,23] to study the relationship between diagnoses. It can also be used for other purposes; for example, to study market baskets [51].

RR is defined based on the diagnoses’ prevalence, as follows:



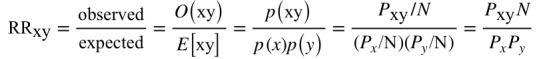



**Figure 1:**
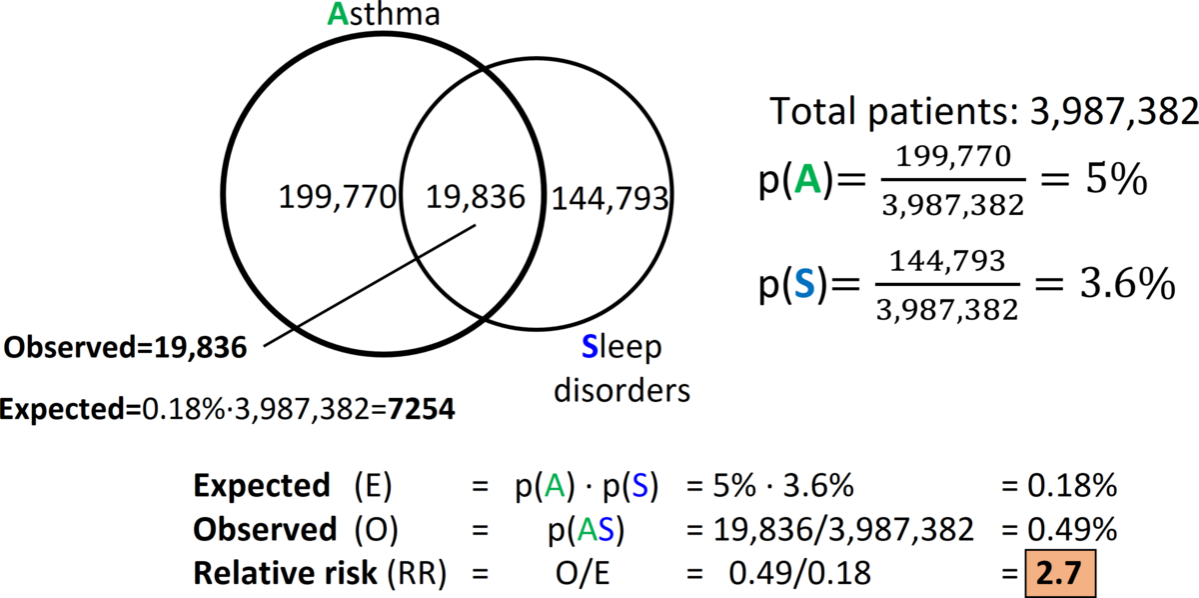
Example of measuring comorbidity by relative risk. Here, asthma and sleep disorders are highly correlated. If they were independent of each other, the probability of a person having both should be p(A) × p(B) = 0.18%, whereas their observed co-occurrence would be 0.49%. Therefore, the relative risk to have both is 2.7 times higher than by random chance.

Here, *p*(*x*) (*P_x_*/N) and *p*(*y*) (*P_y_*/N) are the probabilities that a randomly chosen patient has diseases *x* and *y*, respectively, and *p*(*xy*) (*P_xy_*/N) is the probability that a randomly chosen patient has both diseases. *E*[*xy*] is the expected frequency of *xy*. Figure 1 demonstrates the detailed calculation of the RR values in cases of asthma and sleep disorders. An RR value >1.0 indicates that the 2 diseases are related.

Most RR values are between 0.5 and 5.0; however, they can also be >100. These outlier values would dominate the clustering cost function optimization, and for this reason, we normalized them to the range of (0,1) by using the following variant of the generalized symmetrical sigmoid function [53]:



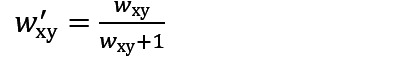



### Multimorbidity Network

**Figure 2:**
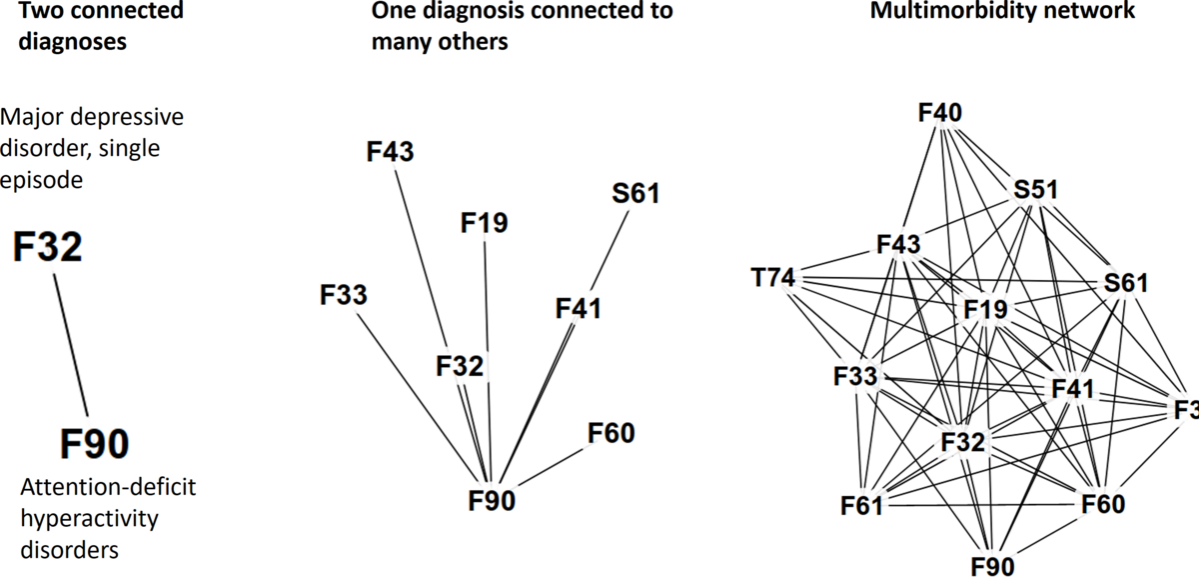
Multimorbidity network formed by finding related diagnoses for all diagnoses in the data set.

A multimorbidity network is formed by connecting all pairs of diagnoses that are related (Figure 2). Each node in this network corresponds to a medical diagnosis, and the strength of the connections can be measured using RR, correlation, or other methods. We used the name multimorbidity network following the choice of Aguado et al [54]. This network has also been called a disease co-occurrence network [48], *phenotypic disease network* [14], *comorbidity network* [17], and *disease comorbidities network* [34].

Several previous studies used multimorbidity networks [14,17,18,34,49,54]. In addition, Klimek et al [55] and Moni and Liò [52] studied comorbidity associations, although they did not explore much of the network analysis. Moni and Liò [52] created R language software called *comoR* for disease comorbidity risk analysis. Divo et al [34] studied chronic obstructive pulmonary disease for disease screening and management. Folino et al [18] predicted future diseases based on past medical history. Srinivasan et al [49] used a multimorbidity network to extract features for a high-cost patient prediction. Hidalgo et al [14] also published multimorbidity network data (based on 13 million patients) [56].

**Figure 3:**
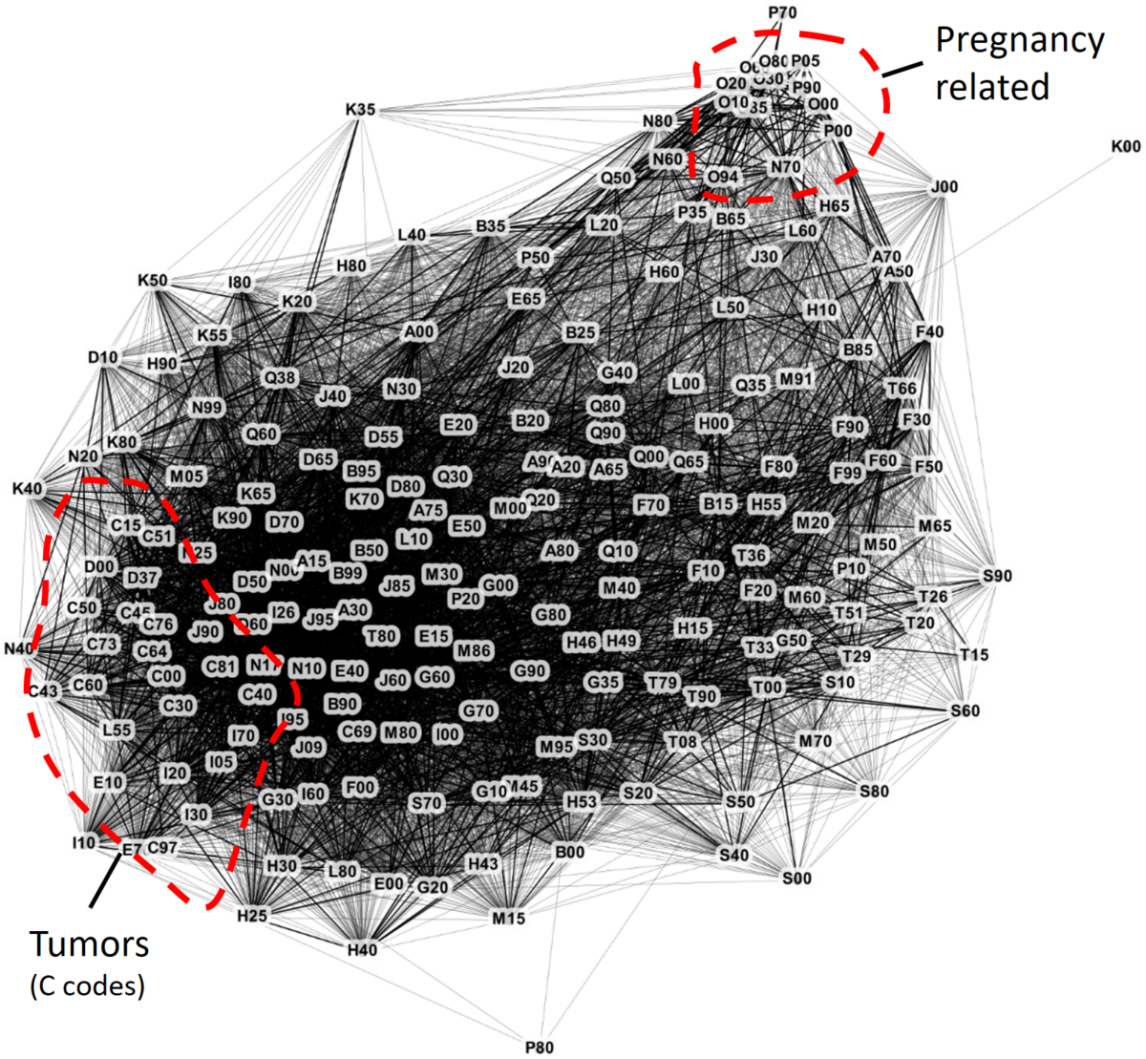
The full network was overwhelming to analyze, with 205 disease subgroups and 14,254 connections overall. Here, we show only the 8895 connections with a relative risk of >1.5. Connections with relative risk >3.0 are drawn in bold. ICD-10 (International Classification of Diseases, 10th revision) subgroups are represented by the first diagnosis of the group (Multimedia Appendix 1). The image was created by using the Gephi software [56]. Only very tight groups such as pregnancy-related diagnoses and tumors can be recognized from the network.

We constructed a multimorbidity network (Figure 3 [57]) by calculating the RR value for all pairs of diagnoses, including those with an RR value ≥1.0 and at least 10 patients with both diagnoses. The accuracy used for diagnoses was the subgroup of the ICD-10 classification (eg, I20-I25). We also filtered out the diagnoses that indicated symptoms and external causes (those starting with Z, W, Y, and R). After filtering, we obtained 205 disease subgroups in the graph (see Multimedia Appendix 1 for the full list).

### Clustering

#### Overview

The main motivation for clustering is that the multimorbidity network is too large (205 nodes and 14,254 connections) for detailed analysis. For this reason, we clustered the graph to form more compact entities of related diseases. The goal was to assign strongly related diseases to the same cluster but keep uncorrelated diseases in different clusters. To achieve this goal, an evaluation criterion was necessary to measure the effectiveness of clustering.

#### Cost Function

Instead of using heuristic criteria such as average or complete linkage, it is better to define an exact cost function that the clustering algorithm optimizes directly. When clustering numerical data, a typical goal is to measure the compactness of the clusters. For example, both the Ward method and k-means minimize the *sum of squared distances* between the data objects to the cluster mean (*centroid*). However, calculating the mean of a subgraph is not possible directly but would require an indirect solution such as vectorizing the nodes by graph embedding [58]. Moreover, calculating the distance between 2 nodes is not possible if they are not connected. Therefore, graph-specific cost functions have been developed to overcome these issues.

Three cost functions were evaluated in the study by Sieranoja and Fränti [26] with controlled data—*conductance*, *mean internal weight*, and *IIW*. The last function produced the most accurate clustering result with balanced cluster sizes and was therefore chosen in this study as well. When *k* is the number of clusters, *W_i_* is the internal weight of cluster *i*, and *M* is the total weight (mass) of the entire graph, the cost is calculated as follows:



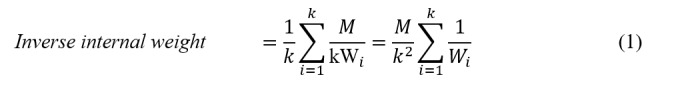



In multimorbidity network analysis, it is desirable to have clusters of approximately the same size. This could be controlled by specifying the number of clusters. As the cost function induces balanced cluster sizes, we aimed to group N nodes into *k* clusters of size N/*k*=n. In our case, we had N=205 diseases and *k*=15 clusters with 205/15=13.7 diseases, on average. This size was sufficiently small to allow us to investigate the clusters manually.

#### Clustering Algorithm

**Figure 4:**
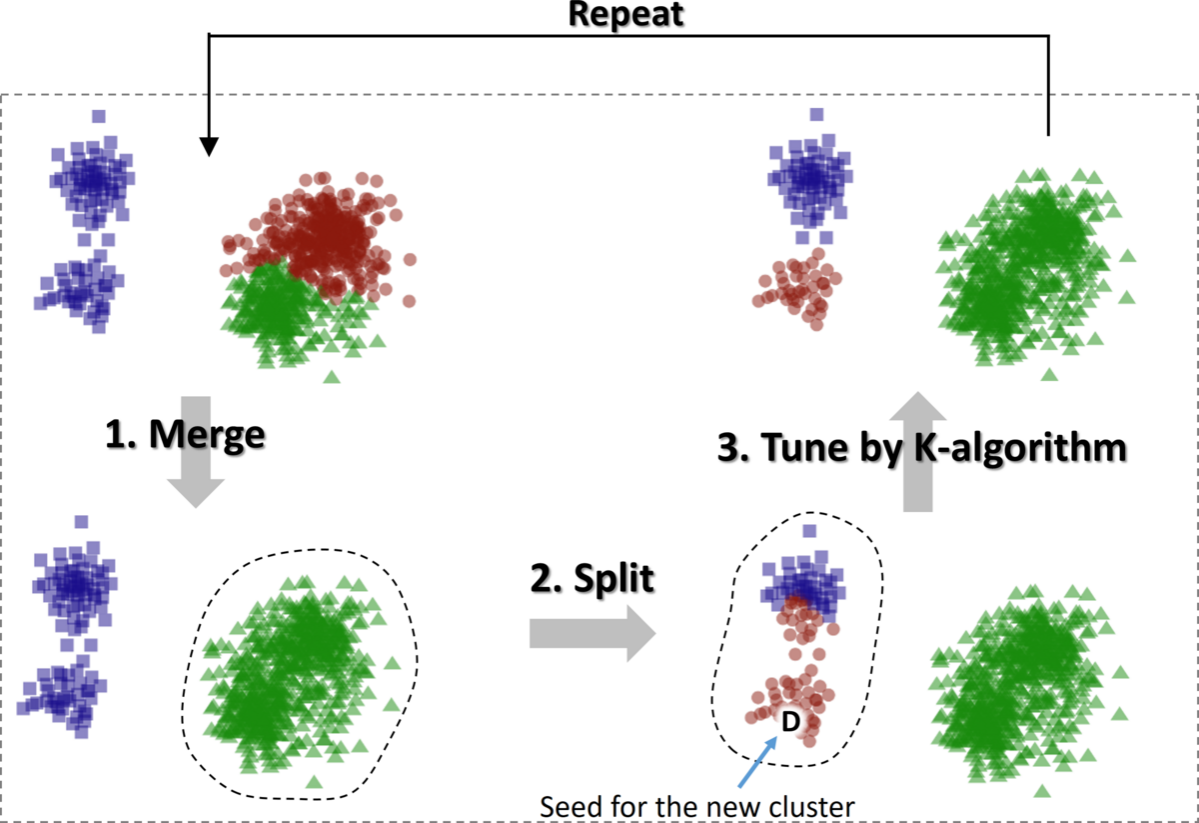
The M-algorithm merges 2 random clusters, splits 1 random cluster, and fine-tunes the result by using the K-algorithm. The network in this example is the k-nearest neighbors graph of the presented 2D data set.

**Figure 5:**
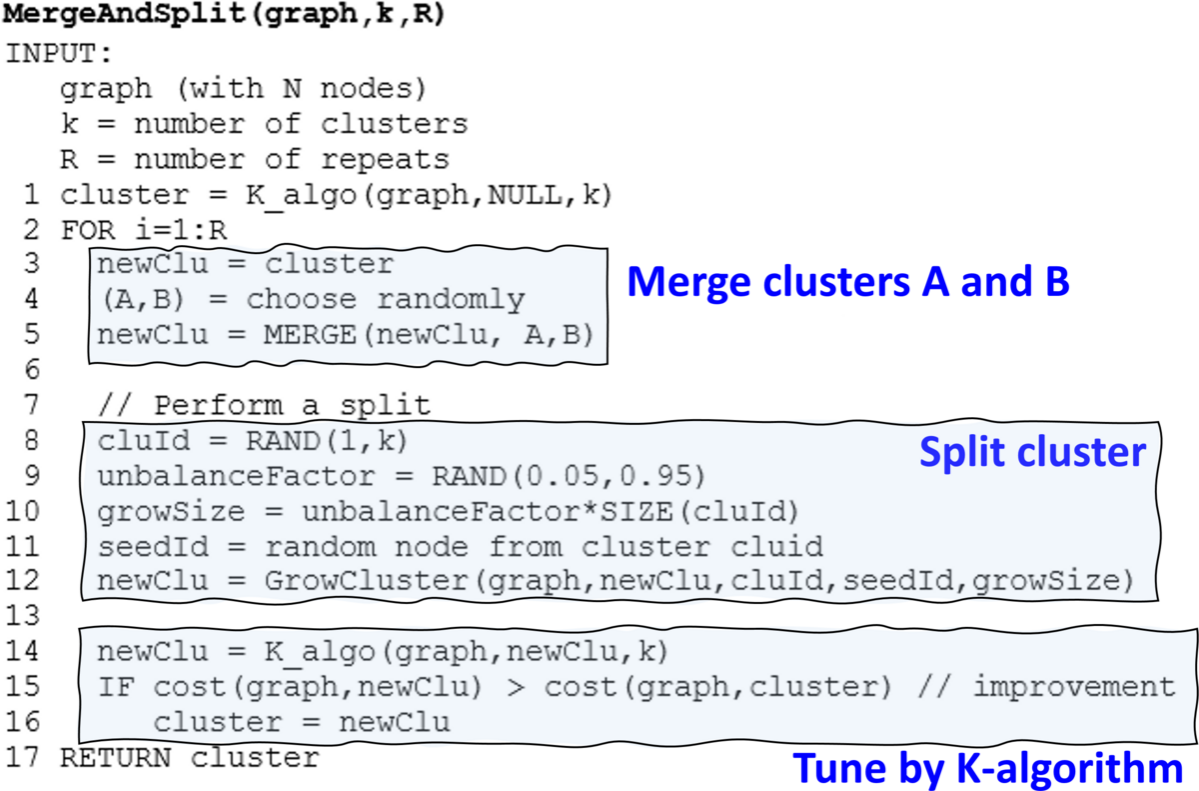
Pseudocode for the M-algorithm.

We used the recently developed M*-algorithm* in [26], which combines a k-means type of iterative optimization with an additional merge and split strategy to escape from local minima (Figures 4-5). The *IIW* was the recommended cost function.

K-means uses two optimization steps: assignment and centroid steps. In the assignment step, every point is placed in the cluster whose mean (centroid) is closest. However, the assignment of points is not independent of the assignment of other points. Their joint effect may cause the cost value to fluctuate so that the total value increases even if the single assignment decreases. To avoid this problem, we used the sequential variant of k-means, where every assignment has an immediate effect on the centroids. This technique prevents fluctuations.

The k-means variant applied to graphs is called the *K-algorithm,* which is similar to the original k-means algorithm but without centroids. The distance calculations were replaced by directly evaluating the effect of the assignment on the cost function. Most cost functions are based on maximizing the weights inside the cluster or minimizing external weights. Therefore, the effect of a node joining a cluster can be calculated using only its edges and the size of the cluster.

The K-algorithm iteratively improves the initial solution by sequentially processing the nodes in random order. For each node, the method considers all clusters and checks whether changing the partition of the node improves the cost function. If it does, the cluster assignment is changed. After all the nodes have been processed, the algorithm starts another iteration. The iterations continue until no changes occur.

The M-algorithm differs from the K-algorithm in the additional merge and split step. The M-algorithm first merges 2 random clusters and then splits 1 random cluster. The clustering solution is fine-tuned using the K‑algorithm. If the new solution improves the cost function value, it is kept as the current solution; otherwise, the process continues from the previous solution. The merge and split process is repeated depending on the amount of computation time required. The pseudocode for the algorithm is presented in Figure 5.

As the network itself is quite small (205 diagnoses), the clustering algorithm takes only a little time. The time complexity of the M-algorithm is *O(RIN[k+|E|/N])*, where *R* is the number of repeats, N is the number of diagnoses (nodes), *k* is the number of clusters, |*E*|/*N* is the average number of connections for each node (diagnosis), and *I* is a small number that reflects the number of iterations to converge. We ran the M-algorithm for 20,000 repeats, which took 27 minutes (single thread) on an Intel Xeon(R) W-2255 CPU at 3.70 GHz. The bottleneck was the *O(N_v_)* network construction, which needed to process all N*_v_*=58 million patient visits and took 52 minutes.

The number of clusters, *k,* must be fixed by the researcher beforehand. A small number is likely to generate large mixed clusters of many diseases, thereby losing the capability to make meaningful observations. A large number of clusters tend to mainly cluster diseases from the same ICD group, which might lose the chance to detect relevant multimorbidity patterns. We tried clustering with several different *k* values and chose *k*=15 as it produced clusters of convenient size for analysis in the form of similarity matrices.

It is also possible for the algorithm to recommend the number of clusters using a suitable cluster validity index that measures the ratio of within-cluster and between-clusters similarities, as in the study by Zhao and Fränti [59]. Wartelle et al [23] derived a validity index from RR and obtained *k*=16 clusters in their data. We used the *silhouette coefficient* [60] for our data, and in the range of 5 to 25, it obtained *k*=17 clusters. They are both close to our choice of *k*=15.

### Ethics Approval

Permission to use the register data was obtained from the Finnish Institute for Health and Welfare. All methods were carried out in accordance with relevant guidelines and regulations or declaration of Helsinki. The Finnish legislation (Act 552/2019) do not require informed consent for register-based research when study is solely based on registers and the study is considered to be of public health importance.

## Results

### RR Measurements

Table 3 shows the 10 pairs of disease subgroups with the highest RR values. They are diagnoses with the highest probability of appearing jointly relative to the expected probability with the independent assumption. Some connections are obvious, often representing the same or closely related conditions (C40-C41 and C45-C49). Some have known explanations in medical science (F70-F79 and Q90-Q99) or a clear causal relationship (D80-D89 and N00-N08). There are also connections with smaller RR values that are not so obvious at first sight; however, they are clinically meaningful (I26-I28 and M30-M36). In addition to using the ICD-10 subgroups, we calculated the RR values for diagnoses with 3-character precision. Some RR values <1.0 were also found for diagnoses such as E10 and E11, which are exclusive to each other.

**Table 3:** The 10 disease pairs with the highest relative risk (RR) valuea.

Diagnosis A		Diagnosis B			RR		Count (n=3987, 382%), %
Code	Description	Code	Description				
A80-A89	Viral infections of the central nervous system	G00-G09	Inflammatory diseases of the central nervous system	170.1		484 (0.01)	
A15-A19	Tuberculosis	B90-B94	Sequelae of infectious and parasitic diseases	110.7		132 (0.00)	
C40-C41	Malignant neoplasms of bone and articular cartilage	C45-C49	Malignant neoplasms of mesothelial and soft tissue	98.3		107 (0.00)	
T20-T25	Burns and corrosions of external body surface, specified by site	T29-T32	Burns and corrosions of multiple and unspecified body regions	91.0		893 (0.02)	
F70-F79	Mental retardation	Q90-Q99	Chromosomal abnormalities, not elsewhere classified	79.7		945 (0.02)	
G35-G37	Demyelinating diseases of the central nervous system	H46-H48	Disorders of optic nerve and visual pathways	50.7		811 (0.02)	
D80-D89	Certain disorders involving the immune mechanism	N00-N08	Glomerular diseases	47.2		2386 (0.06)	
J85-J86	Suppurative and necrotic conditions of lower respiratory tract	J90-J94	Other diseases of pleura	45.7		866 (0.02)	
N25-N29	Other disorders of kidney and ureter	Q60-Q64	Congenital malformations of the urinary system	45.3		328 (0.01)	
F70-F79	Mental retardation	Q00-Q07	Congenital malformations of the nervous system	42.0		238 (0.01)	

^a^Full list is available on the University of Eastern Finland website [27].

### Clustering Results

**Figure 6:**
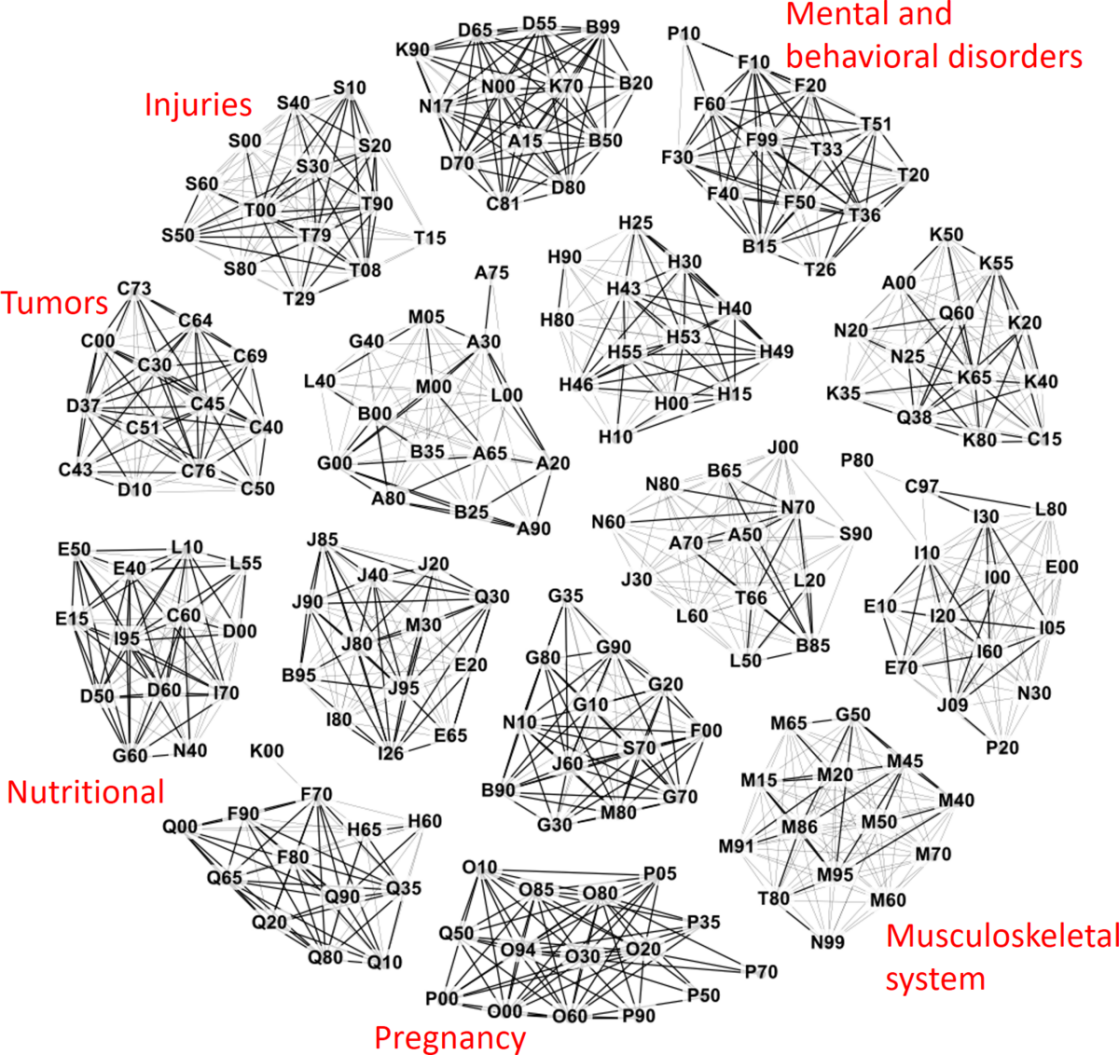
Clusters obtained from the multimorbidity network. Subjective labels of 6 clusters are also shown. This figure shows all 205 diagnoses and only those 1144 connections with relative risk ≥1.5. Cases with a relative risk of ≥3 are shown with thicker lines. International Classification of Diseases, 10th Revision, blocks are represented by the first diagnosis of the block (eg, F10-F19 by F10).

The overall clustering results are visualized as a graph in Figure 6. The graph shows connections within the clusters; however, all connections between clusters have been eliminated for clarity.

**Figure 7:**
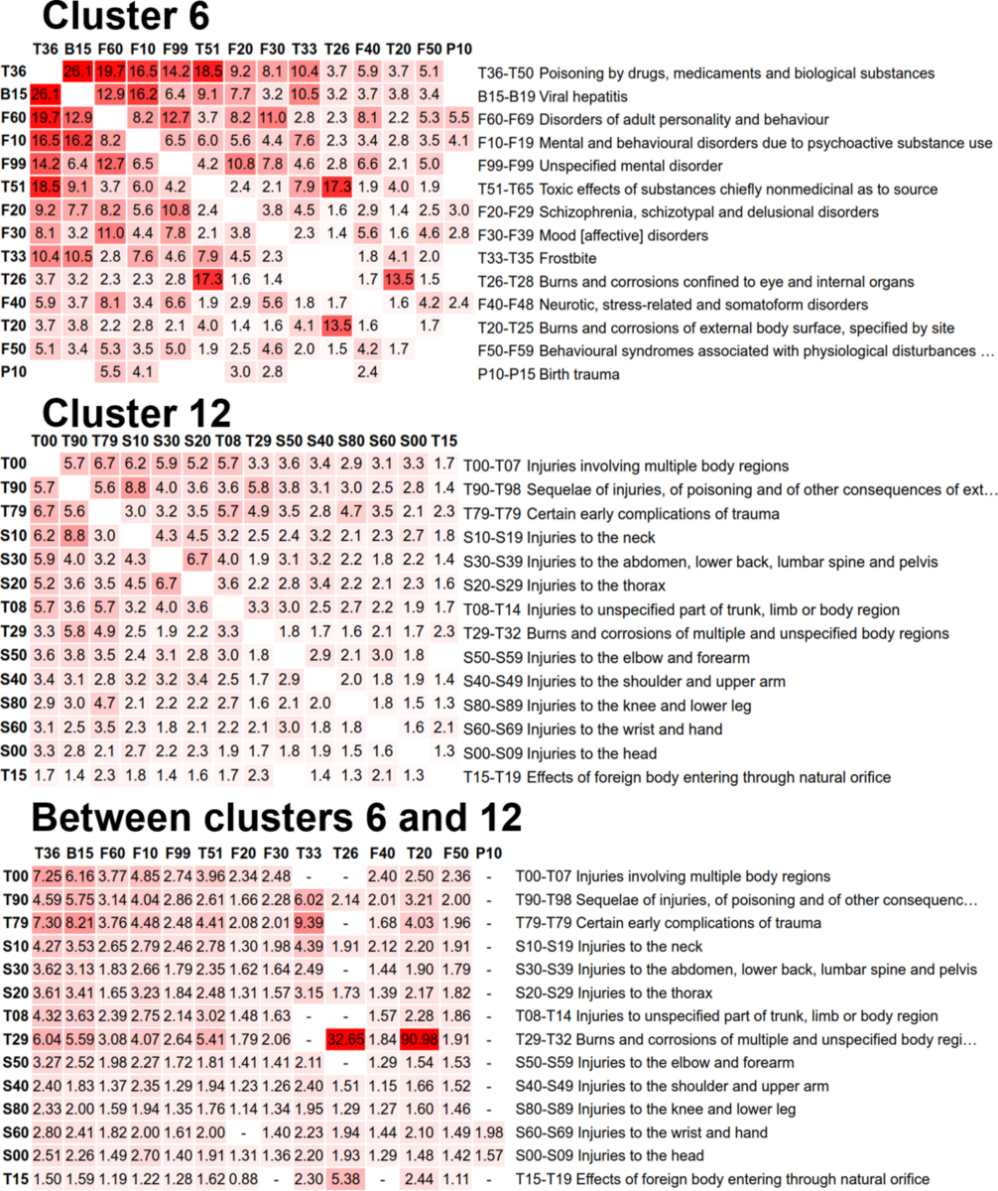
Two example clusters and their connections in between. The numbers are relative risk values. High values and the red color signify stronger relationships. The blocks are represented by the first diagnosis code (eg, T36 represents block T36-T50).

We fixed the number of clusters to 15 for the M-algorithm [26]. This roughly matches the number 16 used in a study by Wartelle et al [23]. The main characteristics of the resulting clusters are summarized in Tables 4 and 5. The strength of the associations between the diagnosis subgroups inside the 2 example clusters and the connections between the 2 clusters can be observed in Figure 7. The number of patients in each cluster, the number of visits to health services, total costs, cost per visit, and cost per patient are reported in Table 6.

**Table 4:** Content of the 15 clusters (ICD-10a blocks) and their strengths as the mean RRb values of diagnoses within the cluster.

Cluster	RR, mean	ICD-10 codes
Cluster 1	11.3	O85-O92; O30-O48; O20-O29; O10-O16; O60-O75; O94-O99; O80-O84; P05-P08; P00-P04; O00-O08; P35-P39; P90-P96; Q50-Q56; P70-P74; P50-P61
Cluster 2	8.1	B50-B64; N00-N08; D70-D77; C81-C96; D55-D59; D80-D89; D65-D69; B99-B99; A15-A19; N17-N19; B20-B24; K70-K77; K90-K93
Cluster 3	7.8	F70-F79; Q90-Q99; F80-F89; Q00-Q07; Q35-Q37; Q80-Q89; Q65-Q79; F90-F98; Q20-Q28; Q10-Q18; H65-H75; H60-H62; K00-K14
Cluster 4	7.6	C40-C41; C45-C49; C76-C80; C30-C39; D37-D48; C69-C72; C00-C14; C51-C58; C64-C68; C73-C75; C50-C50; C43-C44; D10-D36
Cluster 5	5.7	J95-J99; J85-J86; J90-J94; J80-J84; Q30-Q34; I26-I28; J40-J47; B95-B98; M30-M36; J20-J22; E65-E68; E20-E35; I80-I89
Cluster 6	5.4	T36-T50; B15-B19; F60-F69; F10-F19; F99-F99; T51-T65; F20-F29; F30-F39; T33-T35; T26-T28; F40-F48; T20-T25; F50-F59; P10-P15
Cluster 7	4.8	E40-E46; E50-E64; D60-D64; I95-I99; D50-D53; L55-L59; D00-D09; E15-E16; G60-G64; I70-I79; L10-L14; C60-C63; N40-N51
Cluster 8	4.6	G80-G83; G10-G14; J60-J70; G90-G99; F00-F09; G70-G73; G30-G32; N10-N16; B90-B94; S70-S79; M80-M85; G20-G26VG35-G37
Cluster 9	4.5	Q60-Q64; N25-N29; K65-K67; Q38-Q45; C15-C26; K80-K87; K55-K64; K40-K46; N20-N23; K20-K31; K50-K52; A00-A09; K35-K38
Cluster 10	4.3	G00-G09; A80-A89; A90-A99; A65-A69; M00-M03; A30-A49; B25-B34; A20-A28; M05-M14; B00-B09; L00-L08; L40-L45; B35-B49; G40-G47; A75-A79
Cluster 11	3.8	H53-H54; H46-H48; H55-H59; H49-H52; H43-H45; H30-H36; H15-H22; H40-H42; H25-H28; H00-H06; H10-H13; H90-H95; H80-H83
Cluster 12	3.0	T00-T07; T90-T98; T79-T79; S10-S19; S30-S39; S20-S29; T08-T14; T29-T32; S50-S59; S40-S49; S80-S89; S60-S69; S00-S09; T15-T19
Cluster 13	2.9	M95-M99; M40-M43; M45-M49; M86-M90; T80-T88; G50-G59; M15-M19; M20-M25; M50-M54; M91-M94; M65-M68; M70-M79; N99-N99; M60-M63
Cluster 14	2.9	A50-A64; A70-A74; B85-B89; N70-N77; B65-B83; T66-T78; L50-L54; L20-L30; N80-N98; L60-L75; J30-J39; N60-N64; J00-J06; S90-S99
Cluster 15	2.1	I30-I52; I20-I25; I60-I69; I10-I15; L80-L99; I05-I09; J09-J18; E70-E90; N30-N39; E10-E14; E00-E07; I00-I02; P20-P29; C97-C97; P80-P83

^a^ICD-10: International Classification of Diseases, 10th Revision.

^b^RR: relative risk.

**Table 5:** Summarization of the cluster content with their age and gender distributions.

Cluster	Dominant gender			Age (years), median		Age ≥70 years, %		Description
	Gender	Values, n (%)						
Cluster 1: pregnancy	Women	219,566 (99.68)	33		0		Pregnancy, childbirth and the puerperium (O codes), certain conditions and disorders originating in perinatal period (P05-P08, P00-P04, P35-P39, P90-P96, P70-P74, and P50-P61), and congenital malformations of genital organs (Q50-56)	
Cluster 2: immune system and blood-forming organs	Men	110,157 (50.79)	69		50		Infectious diseases strongly affecting the immune system (B50-B64, B20-24, B99-B99, and A15-19); malignant neoplasms of lymphoid, hematopoietic, and related tissue (C81-96); diseases of the kidneys (N00-N08 and N17-N19), liver (K70-77), blood, and blood-forming organs and disorders of the immune mechanism (D70-D77, D55-D59, D80-D89, and D65-D69 [except nutritional and aplastic and other anemias]); and other diseases of the digestive system (K90-K93)	
Cluster 3: mixed cluster; includes mental disorders, malformations, and ear and oral cavity diseases	Women	1,062,480 (55.13)	49		17		Mental retardation (F70-79) and disorders of psychological development or unspecified disorder (F80-F89, F99-F99) and congenital malformations (Q codes except for codes for congenital malformations of the respiratory system, digestive system, genital organs, and urinary system); diseases of the ear (H65-H75 and H60-H62); and diseases of the oral cavity, salivary glands, and jaws (K00-K14)	
Cluster 4: tumors	Women	317,372 (62.64)	66		42		Malignant neoplasms (all C codes, except codes for malignant neoplasms in digestive organs; male genital organs; lymphoid, hematopoietic, and related tissue; multiple independent sites) and benign neoplasms (D10-D36)	
Cluster 5: lower respiratory system	Women	437,591 (59.13)	64		38		Lower respiratory tract diseases and related inflammatory conditions (J95-J99, J85-J86, J90-J94, J80-J84, J40-J47, and J20-J22); congenital malformations of the respiratory system (Q30-Q36), pulmonary heart disease and diseases of pulmonary circulation (I26-I28); bacterial, viral, and other infectious agents (B95-B98); systemic connective tissue disorders (M30-M36), obesity (E65-E68) and disorders of other endocrine glands (E20-E35); and diseases of veins, lymphatic vessels, and lymph nodes not classified elsewhere (I80-I89)	
Cluster 6: mental and behavioral disorders	Women	369,203 (58.30)	46		15		Mental and behavioral disorders and substance abuse problems (F60-F69, F10-F19, F20-F29, F30-F39, F40-F48, F50-F59, and F99); poisonings (T36-T50 and T51-T65) and certain viral infections (B15-B19); and related burns (T20-T25 and T26-T28), frostbite injuries (T33-T35), and birth trauma (P10-P15)	
Cluster 7: nutritional	Men	314,390 (66.98)	72		58		Malnutrition (E40-E46) and nutritional deficiencies (E50-64); anemias (D50-D53 and D60-D64); other and unspecified disorders of the circulatory system (I95-I99); certain skin diseases (L55-L59 and L10-L14); in situ neoplasms (D00-D09); other disorders of glucose regulation and pancreatic internal secretion (E15-E16); polyneuropathies (G60-G64); diseases of arteries, arterioles, and capillaries (I70-I79); and diseases and malignant neoplasms of male genital organs (C60-C63 and N40-N51)	
Cluster 8: diseases related to aging	Women	242,917 (59.83)	76		64		Cerebral palsy, memory disorders, other diseases of the central nervous system or neurodegenerative diseases (included G-codes), lung diseases because of external agents (J60-J70), organic mental disorders (F00-F09), renal tubulointerstitial diseases (N10-N16), changes in bone structure (M80-85) and injuries (hip and thigh S70-S79), and other infections (B90-B94)	
Cluster 9: mixed cluster; includes organ malformations and digestive system disorders	Women	387,222 (54.07)	63		37		Congenital malformations of the urinary system and digestive system (Q60-Q64 and Q38-Q45), some disorders of the kidney and ureter (N25-N29) and genitourinary system (N20-N23), diseases of the digestive system (all K codes, except diseases of the oral cavity, salivary glands and jaw, and diseases of the liver), malignant neoplasms of digestive organs (C15-C26), and intestinal infectious diseases (A00-A09)	
Cluster 10: infections and inflammation	Women	483,595 (53.55)	61		33		Inflammatory diseases (G00-G09)/viral infections (A80-A89) of the central nervous system, hemorrhagic fevers (A90-A99), certain other infectious and parasitic diseases (A65-A69, A30-A49, A20-A28, A75-A79, B00-B09, and B35-B49), infectious arthropathies/inflammatory polyarthropathies (M00-M03 and M05-M14), infections of the skin and subcutaneous tissue or papulosquamous disorders (L00-L08 and L40-L45), and episodic and paroxysmal disorders (G40-G47)	
Cluster 11: eye and ear	Women	491,892 (58.89)	67		45		Diseases of the eye and adnexa (all H codes) and diseases of the inner ear (H80-H83) and other disorders of the ear (H90-H90)	
Cluster 12: injuries	Men	516,849 (51.27)	55		26		Injuries in different parts of the body (all S codes, except injuries to the hip and thigh) and in multiple body regions (T00-T07) or unspecified parts (T08-T14 and T29-T32), effects of foreign bodies entering through a natural orifice (T15-T19), and some of their consequences (T79-T79 and T90-T98)	
Cluster 13: musculoskeletal system	Women	855,218 (58.69)	60		31		Diseases of the musculoskeletal system and connective tissue (all M codes, except infectious and inflammatory arthropathies or poly arthropathies, systemic connective tissue disorders, and disorders of bone density and structure); complications of surgical and medical care (T80-T88); nerve, nerve root, and plexus disorders (G50-G59); and other disorders of the genitourinary system (N99-N99)	
Cluster 14: mixed cluster; includes sexually transmitted, parasitic, and urinary tract diseases	Women	844,339 (65.79)	48		19		Sexually transmitted diseases (A50-A64 and A70-A74), parasitic diseases (B85-B89 and B65-B83), unspecified effects of external causes (T66-T78), inflammatory diseases of female pelvic organs (N70-N77), disorders of the breast (N60-N64), noninflammatory disorders of the female genital tract (N80-N98), some diseases of the skin (L50-L54, L20-L30, and L60-L75), acute and some other upper respiratory infections (J30-J39 and J00-J06), and injuries to the ankle and foot (S90-S99)	
Cluster 15: cardiovascular and metabolic	Women	867,133 (56.22)	68		47		Diseases of the circulatory system (all I codes, except pulmonary heart disease and diseases of pulmonary circulation [I26-I28] and diseases of arteries and veins [I70-I79, I 80-I89, and I95-I99]), other disorders of the skin and subcutaneous tissue (L80-L99), influenza and pneumonia (J09-J18), metabolic disorders (E70-E90), disorders of the thyroid gland (E00-E07), diabetes mellitus (E10-E14), other diseases of urinary system (N30-N39), respiratory and cardiovascular disorders specific to the perinatal period (P20-P29), malignant neoplasms of independent (primary) multiple sites (C97-C97), and conditions involving the integument and temperature regulation of fetus and newborn (P80-P83)	

**Table 6:** Estimated (annual) costs of each clustera.

Cluster	Description	Patients^b^ (n=2,536,944), n (%)	Visits^b^ (n=14,597,901), n (%)	Total cost^b^ (€^c^; millions)	Cost per visit^b^ (€)	Cost per patient^b^ (€)
1	Pregnancy	78,159 (3.08)	255,902 (1.75)	207	810	2648
2	Immune system and blood-forming organs	95,865 (3.78)	653,500 (4.48)	521	798	5435
3	Mental disorders, malformations, ear and mouth	838,208 (33.04)	1,899,209 (13.01)	324	171	387
4	Tumors	210,272 (8.29)	1,046,147 (7.17)	704	673	3348
5	Lower respiratory system	299,482 (11.80)	953,199 (6.53)	620	651	2070
6	Mental and behavioral disorders	280,450 (11.05)	2,094,496 (14.35)	908	434	3238
7	Nutritional	194,250 (7.66)	708,930 (4.86)	525	741	2703
8	Diseases related to aging	172,194 (6.79)	1,105,325 (7.57)	730	661	4239
9	Organ malformations and digestive system	262,362 (10.34)	867,971 (5.95)	720	829	2744
10	Infections and inflammation	359,738 (14.18)	1,110,728 (7.61)	627	564	1743
11	Eye and ear	320,947 (12.65)	827,680 (5.67)	298	359	929
12	Injuries	324,191 (12.78)	720,282 (4.93)	417	579	1286
13	Musculoskeletal system	616,550 (24.30)	1,704,486 (11.68)	836	490	1356
14	Sexually transmitted, parasitic, urinary tract	474,604 (18.71)	955,465 (6.55)	290	303	611
15	Cardiovascular and metabolic	773,406 (30.49)	3,326,018 (22.78)	2258	679	2920

^a^A patient and a visit can belong to multiple clusters. Visits and costs include only visits and related costs for diagnoses in a cluster. The cost per visit is calculated as an average for the whole 4-year period; all other values are annual.

^b^Number of patients: mean 353,378; number of visits: mean 1,215,289; cost: mean €666 million; cost per visit: mean €583; cost per patient: mean €2377.

^c^A currency exchange rate of €1=US $1.09 is applicable.

Most clusters were dominated by records of female patients. Cluster 1 (219,566/220,280, 100%) included only women, as it comprised pregnancy-related diagnoses. Other clusters with >60% of records of women were cluster 14 (844,339/1,283,478, 65.7%) of mixed diseases (sexual and urinary) and cluster 4 (317,372/506,660, 62.6%) of malignant tumors. The only cluster with a significantly higher proportion of diagnoses from men was cluster 7 (314,390/469,378, 66.9%), which comprised diagnoses mainly related to nutrition. In most other clusters, the proportions of men and women were approximately equal.

The main reasons for female dominance were that the full database included 1,999,325 men and 2,253,669 women and that women had an average of 6.6 diagnoses, whereas men had only 5.4 diagnoses. A possible reason is that there is a lower threshold for women to seek help from health services than for men. For example, the study by Corrigan [61] suggested that social factors discourage men from seeking mental health care, which can lead to the absence of mental health–related multimorbidities among men.

As all diagnoses were forced to belong to a cluster, there were several mixed clusters. For example, the largest cluster (cluster 3) comprised 33.04% (838,208/2,536,944) of patients, including those with dental health problems (K00-K14). If this subgroup of diagnoses were removed, the number of patients would decrease to only 87,634 and would mainly comprise diagnoses related to mental retardation, congenital malformations, and chromosomal abnormalities. However, it is quite logical that dental health–related diagnoses are clustered with mental retardation; congenital malformations; and abnormalities, such as patients with malformations in the oral cavity, jaws, and teeth, which is a patient group treated in the public health service system.

The second-largest cluster (cluster 15), comprising 30.49% (773,406/2,536,944) of patients, included cardiovascular, endocrine, and metabolic diseases. It also had the highest number of visits to health care (3.3 million annual visits). The third-largest cluster (cluster 13) had 24.30% (616,550/2,536,944) of patients but was more focused on diagnoses related to diseases of the musculoskeletal system and connective tissues. Other more clearly focused clusters included tumors (cluster 4), mental disorders (cluster 6), injuries (cluster 12), diseases related to nutrition (cluster 7), and pregnancy (cluster 1). These clusters can be easily explained based on morbidity and mortality data in Finland. Cardiovascular diseases are still the major cause of death [62], and mental disorders are the main cause of disability pensions, followed by musculoskeletal disorders [63].

These clusters also had clear age profiles. The average age of most clusters was rather high, being ≥60 years in the case of 10 clusters. The exceptions were cluster 6 (mental; mean 46 years), cluster 12 (injuries; mean 55 years), mixed clusters 3 (mental, ear, and oral cavity; mean 49 years) and 14 (sexual and urinary; mean 48 years), and cluster 1 (pregnancy; mean 33 years).

**Figure 8:**
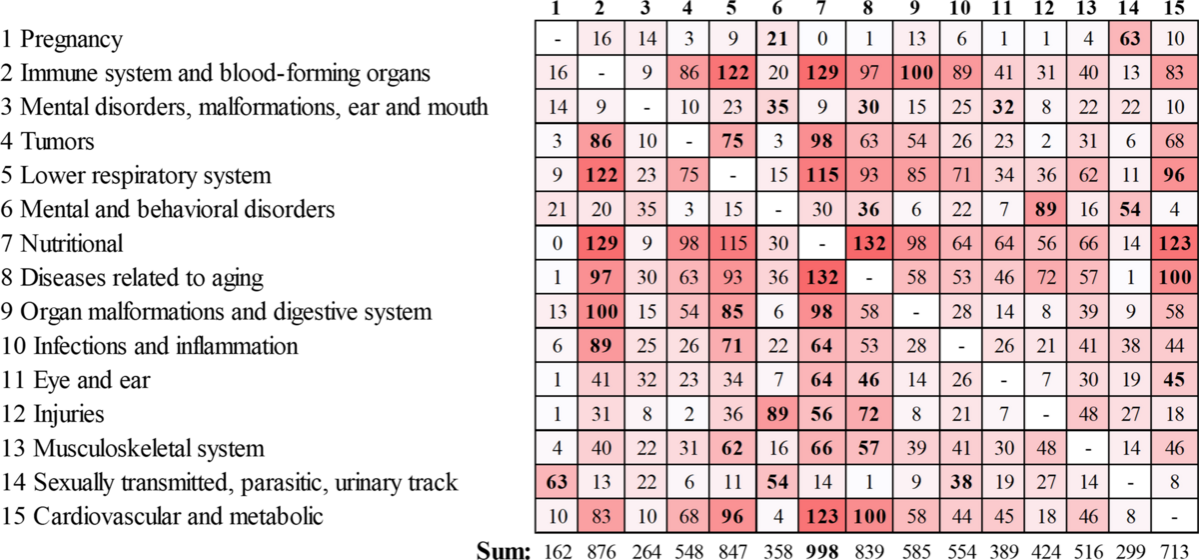
Connections between clusters. Each cluster is represented in the rows with the number and description and in the columns with the number. Values in the table represent the number of links with a relative risk of >2.0 between the clusters. Higher values signify a stronger connection and are emphasized by the red color. Three clusters with the highest values for each row are highlighted with bold font.

Although clustering captures many connections between diseases, it does not capture all information. In fact, many interesting connections can be found by analyzing how strongly the clusters are connected to each other (Figure 8). Cluster 7 (nutritional problems) was the most central cluster, with a strong connection to 10 other clusters. Cluster 1 (pregnancy) was also connected to cluster 6 (mental and behavioral disorders). For example, pregnancy with abortive outcomes (O00-O08) had 5 connections with RR >2 to cluster 6 (mental and behavioral disorders), including neurotic, stress related, mood disorders, and drug poisoning (T36-T50).

Cluster 12 (injuries) had strong connections with clusters 6, 7, and 8. For example, the connection to the nutritional problems cluster had 56 links, with an RR >2. Of these links, 9 came from connections to other and unspecified disorders of the circulatory system (I95-I99).

Figure 7 shows the connections between clusters 6 and 12 in more detail. Cluster 6 comprised mental health (eg, F30-F39 and F60-F69) and substance abuse–related (T36-T50 and F10-F19) diagnoses. Cluster 12 comprised fractures and other injuries. These clusters had a strong connection. A possible explanation is that mental health and substance abuse problems often lead to painful, fracture-causing accidents.

### Cost Effect

The costs of all visits, ward stays, and other contacts of patients belonging to the cluster were calculated for those contacts in services with a diagnosis belonging to the cluster. The estimated costs for each cluster are presented in Table 5. The costs are in euro currency (€).

In general, the cost depends on the number of patients and visits. The largest cluster (cardiovascular and metabolic cluster 15) had 3.3 million visits and €2.3 billion in total costs. However, the cost per patient (€2920) was not the highest, and the cost per visit (€679) was only slightly above average. The diseases in the cluster, such as cardiovascular and metabolic disorders, are largely treated in primary health care, and thus, the average visit cost remains relatively low.

For each patient, the highest costs were in cluster 2 (€5435), including infectious diseases strongly affecting the immune system, diseases of the blood and blood-forming organs, and other disorders involving the immune mechanism. These diseases are likely to need frequent contact with specialized care. Per-patient costs were also high in cluster 8 (diseases related to aging), including diagnoses of neurodegenerative diseases and memory disorders requiring frequent health care contacts and intensive care. The cheapest clusters per patient were cluster 3 (mental disorders, malformations, and ear and mouth; €387) and cluster 14 (sexually transmitted, parasitic, and urinary tract diseases; €611). However, if dental diagnoses were removed, the cost for cluster 3 would be €1144.

The highest cost per visit (€829) was in cluster 9, including organ malformations and diseases of the digestive system. The second-highest cost per visit was observed in cluster 1 (pregnancy), where the cost per visit was €810. This is likely because of delivery-related hospital stays, operations, and other specialized care. Regular maternity care visits are not usually recorded using the ICD-10 codes. Clusters with the lowest cost per visit were the same as those with the lowest cost per patient.

Table 7 shows how the costs of some clusters have developed during the years relative to the total cost of all clusters in the same year. Only clusters with a visible trend (increasing or decreasing) are shown. Clusters that included tumors, lower respiratory system, and eye and ear steadily increased their proportion of all costs from 2015 to 2018, as well as the cluster that included inflammatory diseases and infections, among a few others. The diseases included in these clusters increase with age, and thus, the increase in costs is most likely because of the aging of the population.

**Table 7:** Trends of the annual costs (relative to all costs) of selected clusters from 2015 to 2018.

Trend and cluster			2015		2016		2017		2018
**Increasing trend, %**									
	Tumors	7		7.1		7.2		7.4	
	Mixed cluster 10	6.1		6.3		6.4		6.6	
	Lower respiratory system	6.1		6.2		6.4		6.4	
	Eye and ear	2.9		3		3.1		3.2	
**Decreasing trend, %**									
	Mental and behavioral disorders	9.5		9		9		8.8	
	Mixed cluster 8	6.9		6.7		6.7		6.3	
	Injuries	4.4		4.2		4.2		4	
	Pregnancy	2.4		2.2		2		2	

The relative costs of mental and behavioral disorders decreased the most (from 9.5% to 8.8%), whereas injuries (4.4% to 4.0%) and pregnancy-related diseases (2.4% to 2.0%) also showed a clear decrease. There are several explanations for the observed decline in the costs of care related to mental and behavioral disorders, including the current tendency to prefer outpatient services and difficulties in appropriate service provision. The absolute cost values for pregnancy-related issues were €219 million, €213 million, €202 million, and 194 million from 2015 to 2018. Therefore, the decrease is real, which could be explained by the decrease in the birth rate from 1.65 to 1.41 during the same period (1.65, 1.57, 1.49, and 1.41) [64].

## Discussion

### Principal Findings

We analyzed the data by clustering the diagnoses into 15 clusters. All clusters were consistent with expert knowledge of the domain. Some of these clusters were expected. For example, mental and behavioral disorders were so closely associated with substance abuse problems that they formed one cluster. Some clusters also showed interesting and unexpected connections, such as a cluster that included lower respiratory tract diseases and systemic connective tissue disorders. Although some connections are easily justified by the close relation of the diagnoses, they are not necessarily considered when planning the current service processes and resources. For example, understanding the strong connections between many disorders related to aging could improve the treatment processes of older patients who are multimorbid.

Analysis of the connections between clusters also provided interesting details. For example, the mental health and substance abuse cluster was very closely connected to the cluster comprising fractures and other injuries. A possible explanation is that mental health and substance abuse problems often lead to painful, fracture-causing accidents. The nutritional problems cluster was the most central in the data, with a strong connection to 10 other clusters. This is an interesting finding that addresses the connection between nutritional status and various health disorders.

For each patient, the highest costs were in cluster 2 (€5435), which included infectious diseases that strongly affect the immune system, diseases of the blood and blood-forming organs, and other disorders involving the immune mechanism. These diseases are likely to need frequent contact with specialized care.

Clusters associated with an aging population increased their proportion of all costs from 2015 to 2018. These clusters included diseases related to tumors, lower respiratory system, and eye and ear. The relative costs of mental and behavioral disorders decreased the most (from 9.5% to 8.8%), which might be partly explained by the current tendency to prefer outpatient services.

### Limitations

The underlying data reflect how patients use health services and are diagnosed during health care contacts, which may not always accurately reflect the true relationship between diseases. For example, a person who visits health services only for caries treatment may not be as easily diagnosed with alcohol-related disorders (F10) or problems related to metabolic disorders (E66) as a person who visits because of mental health issues or maternity issues.

The clustering methodology itself has a few limitations. Although the chosen clustering algorithm and cost function were shown to have good clustering accuracy with validation data, it forces every diagnosis to belong to a cluster, even if it does not have any connections to other diagnoses. A possible improvement could be the application of outlier detection as a preprocessing step to remove such cases.

Another limitation is that every diagnosis can belong to only one cluster, although it can be connected to diseases in several clusters. For example, dental health diagnoses were clustered with mental retardation and malformations but are clearly very relevant comorbidities for other chronic conditions such as diabetes. In addition, many infectious disease subgroups are likely to have significant connections with many chronic conditions that decrease the immune response, such as tumors.

The data might also be biased by domestic characteristics within the Finnish population and traditions in recording diagnoses. For example, some conditions such as substance abuse disorders are still highly stigmatized and thus underdiagnosed. The research goal was to find relevant multimorbidity diseases that have a high cost effect on the Finnish health care system. Although some bias might exist, we expect most multimorbidity patterns to appear in other high-income countries, and therefore, the main results might be globally generalizable. This finding was partly confirmed by similar studies in the United States [65] and France [23].

Comparison with other clustering results in earlier studies was challenging mainly because there are many variations in the definition and measures of multimorbidity, as well as the data sources, such as registers, health records, and self-reports, which have been used to obtain information on comorbidities. These differences make comparison difficult but still possible to some degree, as shown in the studies by Prados-Torres et al [13] and Wartelle et al [23].

### Comparison With Prior Work

#### Comparison of Clusters

Wartelle et al [23] obtained 16 clusters (vs 15 in our case). Some of these were similar to ours. For example, cluster 5 contained diagnoses related to mental disorders, substance abuse, and fractures. In our results, substance abuse and mental problems also formed a cluster, which was closely connected to another cluster with different types of fractures. Their data also included one women-specific cluster with pregnancy-related diagnoses. However, most of the clusters were very different from ours.

Their clusters were more unbalanced in size; 5 of the clusters contained only 1 diagnosis, and the largest cluster had 13 diagnoses. In our case, the smallest cluster was size 13, and the largest size was 15. This is partly because of our choice of a clustering cost function that favors more balanced clusters and also because the choice of ED data in [23] was expected to generate larger clusters for trauma diagnoses.

Most of the differences originated from the data. Our data are from everyday health care visits, whereas the data studied by Wartelle et al [23] came from ED visits. They had a smaller number of diagnoses (162 vs 205). These included symptom codes (R00-R99) and factors influencing health status (Z00-Z99), which we removed as we found them to confuse the analysis. These data-related factors produced several clear differences in the results, which we report in the following sections.

The first difference from the study by Wartelle et al [23] is that our data had a female majority (2,062,110/3,835,531, 53.7%). We had only 3 clusters with more male than female patients (nutritional 314,390/469,378, 66.9%; injuries 516,849/1,008,118, 51.2%; immune system and blood-forming organs 110,157/216,898, 50.7%). The ED data had 10 clusters with a male majority (52%-64%). A likely explanation is that these clusters were either directly or indirectly related to trauma commonly treated in EDs, whereas our data represent the services used in primary health care, which has only one cluster (cluster 12) related to injuries.

Patients in the ED data were also much younger than those in our data (mean age 40 years vs 51 years). There were 3 clusters in which the average age of patients exceeded 50 years. One of the clusters (approximately 50%) mostly comprised children aged <5 years. Our data were restricted to adult patients. ED data also lacked a clear pregnancy cluster, and pregnancy-related diagnoses were merged with digestive- and menstruation-related diagnoses.

Busija et al [66] conducted a meta-analysis investigating 51 different articles on multimorbidity profiles. They constructed a similarity matrix of health conditions by counting the number of times each pair of diseases appeared within the same group. The similarity matrix was then projected onto a 2D surface using multidimensional scaling (SPSS/PROXSCAL). This was performed separately for 4 different types of studies grouped by methodology: exploratory factor analysis, cluster analysis of diseases, latent class analysis, and cluster analysis of people.

Overall, their data had fewer diagnoses and clusters. The largest case (factor analysis) included only 70 diagnoses, and they manually distinguished 5 clusters (with a group of mental health problems as one axis) from the 2D projection. They reported clustering of vision, hearing impairment, and fractures in 2 of the 4 cases. In our data, vision and hearing problems were in one cluster, and fractures were in another. These were also weakly connected. A mental health group was visible in all 4 cases and was closely associated with addictions. This is consistent with our results, where mental health and substance abuse problems formed 1 cluster.

#### Comparison of Costs

We compared the cost of our data with that reported by the Milken Institute in the United States in 2016 [65]. The costliest (both direct and indirect costs) chronic disease in the United States is diabetes type 2, with direct costs of US $185 billion. When indirect costs are included, the four most costly diseases were hypertension (US $1042 billion), diabetes type 2 (US $526 billion), chronic back pain (US $440 billion), and osteoarthritis (US $430 billion).

The costliest diseases (hypertension and type 2 diabetes) are in accordance with our results, where the costliest is cluster 15 (cardiovascular and metabolic), which includes hypertension and diabetes-related diagnoses (I10-I15 and E10-E14), as well as other related cardiovascular diseases common in the Finnish population. The costs of the cluster become high as the size of the patient population increases, as well as the need for frequent contact with health care, although costs per visit are close to average.

### Conclusions

To the best of our knowledge, this is the first clustering study with such a rich data set, including all health care visits of Finnish adults aged ≥18 years, covering both primary- and secondary-level care. Good coverage is important, as the tendency in the development of health service systems is to seek better integration of services, including the integration of primary health care, specialized care, and social services.

Identifying multimorbidity clusters, related characteristics, and especially the burden they cause for service use and costs is helpful in estimating the resources needed in the service system, including the specialties and other knowledge profiles of professionals. Such information could also be applied to estimate future needs when, for example, the projections of population aging and other demographics are known.

To the best of our knowledge, this is the first study to use k-means–based clustering of diseases. Although the standard k-means algorithm can be unstable, we used a recent modification called the M-algorithm, which was shown to be accurate on controlled validation data sets. This directly optimizes a cost function for a network that has RR values as weights. Existing studies rely mainly on agglomerative clustering, using either a heuristic cost function such as average or complete linkage or a slow calculation of the RR. The methodology used was accurate and scalable for large-scale data.

In a future study, we will consider clustering patients and comparing whether the same diagnoses can be grouped together. Another idea is to study geographical differences within Finland. The data are large, and as they are publicly available, they have a high potential for others to find more interesting results by data mining.

## Appendix

Multimedia Appendix 1 ICD-10 (International Classification of Diseases, 10th Revision) blocks.

## Abbreviations

ED: emergency department
ICD: International Classification of Diseases
ICD-9-CM: International Classification of Diseases, Ninth Revision, Clinical Modification
ICD-10: International Classification of Diseases, 10th Revision
IIW: inverse internal weight
RR: relative risk
TRIPOD: Transparent Reporting of a multivariable prediction model for Individual Prognosis or Diagnosis

## References

[1] van Oostrom SH, Picavet HS, de Bruin SR, Stirbu I, Korevaar JC, Schellevis FG, Baan CA (2014). Multimorbidity of chronic diseases and health care utilization in general practice. BMC Fam Pract.

[2] van den Akker M, Buntinx F, Knottnerus JA (1996). Comorbidity or multimorbidity. Eur J Gen Pract.

[3] van den Akker M, Buntinx F, Metsemakers JF, Roos S, Knottnerus JA (1998). Multimorbidity in general practice: prevalence, incidence, and determinants of co-occurring chronic and recurrent diseases. J Clin Epidemiol.

[4] Willadsen TG, Bebe A, Køster-Rasmussen R, Jarbøl DE, Guassora AD, Waldorff FB, Reventlow S, Olivarius ND (2016). The role of diseases, risk factors and symptoms in the definition of multimorbidity - a systematic review. Scand J Prim Health Care.

[5] Xu X, Mishra GD, Jones M (2017). Evidence on multimorbidity from definition to intervention: an overview of systematic reviews. Ageing Res Rev.

[6] Wang L, Si L, Cocker F, Palmer AJ, Sanderson K (2018). A systematic review of cost-of-illness studies of multimorbidity. Appl Health Econ Health Policy.

[7] Brettschneider C, Leicht H, Bickel H, Dahlhaus A, Fuchs A, Gensichen J, Maier W, Riedel-Heller S, Schäfer I, Schön G, Weyerer S, Wiese B, van den Bussche H, Scherer M, König HH, MultiCare Study Group (2013). Relative impact of multimorbid chronic conditions on health-related quality of life--results from the MultiCare Cohort Study. PLoS One.

[8] Stirland LE, González-Saavedra L, Mullin DS, Ritchie CW, Muniz-Terrera G, Russ TC (2020). Measuring multimorbidity beyond counting diseases: systematic review of community and population studies and guide to index choice. BMJ.

[9] Farley JF, Harley CR, Devine JW (2006). A comparison of comorbidity measurements to predict healthcare expenditures. Am J Manag Care.

[10] Travers DA, Haas SW, Waller AE, Tintinalli JE (2003). Diagnosis clusters for emergency medicine. Acad Emerg Med.

[11] Schneeweiss R, Cherkin DC, Hart LG, Revicki DA, Wollstadt LJ, Stephenson MJ, Froom J, Dunn EV, Tindall HL, Rosenblatt RA (1986). Diagnosis clusters adapted for ICD-9-CM and ICHPPC-2. J Fam Pract.

[12] Jain AK, Dubes RC (1988). Algorithms for clustering data.

[13] Prados-Torres A, Calderón-Larrañaga A, Hancco-Saavedra J, Poblador-Plou B, van den Akker M (2014). Multimorbidity patterns: a systematic review. J Clin Epidemiol.

[14] Hidalgo CA, Blumm N, Barabási AL, Christakis NA (2009). A dynamic network approach for the study of human phenotypes. PLoS Comput Biol.

[15] Estiri H, Klann JG, Murphy SN (2019). A clustering approach for detecting implausible observation values in electronic health records data. BMC Med Inform Decis Mak.

[16] Huang L, Shea AL, Qian H, Masurkar A, Deng H, Liu D (2019). Patient clustering improves efficiency of federated machine learning to predict mortality and hospital stay time using distributed electronic medical records. J Biomed Inform.

[17] Kalgotra P, Sharda R, Croff JM (2017). Examining health disparities by gender: a multimorbidity network analysis of electronic medical record. Int J Med Inform.

[18] Folino F, Pizzuti C, Ventura M (2010). A comorbidity network approach to predict disease risk. Proceedings of the 1st International Conference on Information Technology in Bio- and Medical Informatics.

[19] Folino F, Pizzuti C (2012). Link prediction approaches for disease networks. Proceedings of the 3rd International Conference on Information Technology in Bio- and Medical Informatics.

[20] Ding R, Jiang F, Xie J, Yu Y (2017). Algorithmic prediction of individual diseases. Int J Prod Res.

[21] John R, Kerby DS, Hennessy CH (2003). Patterns and impact of comorbidity and multimorbidity among community-resident American Indian elders. Gerontologist.

[22] Marengoni A, Bonometti F, Nobili A, Tettamanti M, Salerno F, Corrao S, Iorio A, Marcucci M, Mannucci PM, Italian Society of Internal Medicine (SIMI) Investigators (2010). In-hospital death and adverse clinical events in elderly patients according to disease clustering: the REPOSI study. Rejuvenation Res.

[23] Wartelle A, Mourad-Chehade F, Yalaoui F, Chrusciel J, Laplanche D, Sanchez S (2021). Clustering of a health dataset using diagnosis co-occurrences. Appl Sci.

[24] Violán C, Roso-Llorach A, Foguet-Boreu Q, Guisado-Clavero M, Pons-Vigués M, Pujol-Ribera E, Valderas JM (2018). Multimorbidity patterns with K-means nonhierarchical cluster analysis. BMC Fam Pract.

[25] Fränti P, Sieranoja S (2019). How much can k-means be improved by using better initialization and repeats?. Pattern Recognition.

[26] Sieranoja S, Fränti P (2021). Adapting k-means for graph clustering. Knowl Inf Syst.

[27] Multimorbidity network analysis. University of Eastern Finland.

[28] Whitty CJ, Watt FM (2020). Map clusters of diseases to tackle multimorbidity. Nature.

[29] Moons KG, Altman DG, Reitsma JB, Ioannidis JP, Macaskill P, Steyerberg EW, Vickers AJ, Ransohoff DF, Collins GS (2015). Transparent Reporting of a multivariable prediction model for Individual Prognosis or Diagnosis (TRIPOD): explanation and elaboration. Ann Intern Med.

[30] Newman ME (2006). Modularity and community structure in networks. Proc Natl Acad Sci U S A.

[31] Newman ME (2004). Analysis of weighted networks. Phys Rev E.

[32] Kernighan BW, Lin S (1970). An efficient heuristic procedure for partitioning graphs. Bell Syst Tech J.

[33] Shi J, Malik J (2000). Normalized cuts and image segmentation. IEEE Trans Pattern Anal Machine Intell.

[34] Divo MJ, Casanova C, Marin JM, Pinto-Plata VM, de-Torres JP, Zulueta JJ, Cabrera C, Zagaceta J, Sanchez-Salcedo P, Berto J, Davila RB, Alcaide AB, Cote C, Celli BR, BODE Collaborative Group (2015). COPD comorbidities network. Eur Respir J.

[35] Hromic H, Prangnawarat N, Hulpus I, Karnstedt M, Hayes C (2015). Graph-based methods for clustering topics of interest in Twitter. Proceedings of the 15th International Conference on Engineering the Web in the Big Data Era.

[36] Fortunato S (2010). Community detection in graphs. Phys Rep.

[37] Fortunato S, Hric D (2016). Community detection in networks: a user guide. Phys Rep.

[38] Blondel VD, Guillaume JL, Lambiotte R, Lefebvre E (2008). Fast unfolding of communities in large networks. J Stat Mech.

[39] Lancichinetti A, Fortunato S (2009). Benchmarks for testing community detection algorithms on directed and weighted graphs with overlapping communities. Phys Rev E Stat Nonlin Soft Matter Phys.

[40] Whang JJ, Gleich DF, Dhillon IS (2016). Overlapping community detection using neighborhood-inflated seed expansion. IEEE Trans Knowl Data Eng.

[41] Lu Z, Wen Y, Cao G (2013). Community detection in weighted networks: algorithms and applications. Proceedings of the 2013 IEEE International Conference on Pervasive Computing and Communications.

[42] Lancichinetti A, Fortunato S (2009). Community detection algorithms: a comparative analysis. Phys Rev E Stat Nonlin Soft Matter Phys.

[43] Zhang W, Wang X, Zhao D, Tang X (2012). Graph degree linkage: agglomerative clustering on a directed graph. Proceedings of the 12th European Conference on Computer Vision.

[44] LaSalle D, Karypis G (2016). A parallel hill-climbing refinement algorithm for graph partitioning. Proceedings of the 45th International Conference on Parallel Processing.

[45] Tabatabaei SS, Coates M, Rabbat M (2012). GANC: greedy agglomerative normalized cut for graph clustering. Pattern Recognit.

[46] Mustonen E, Hörhammer I, Absetz P, Patja K, Lammintakanen J, Talja M, Kuronen R, Linna M (2020). Eight-year post-trial follow-up of health care and long-term care costs of tele-based health coaching. Health Serv Res.

[47] Linna M, Mikkola T, Peltokorpi A, Tyni T (2016). Rekistereistä tietoa vanhuspalveluiden johtamiseen? Ikääntyneen väestön sosiaali- ja terveyspalveluiden käytön arviointi rekisteriaineistoja hyödyntämällä.

[48] Kapiainen S, Väisänen A, Haula T (2014). Terveyden- ja sosiaalihuollon yksikkökustannukset Suomessa vuonna 2011.

[49] Srinivasan K, Currim F, Ram S (2018). Predicting high-cost patients at point of admission using network science. IEEE J Biomed Health Inform.

[50] Cornell JE, Pugh JA, Williams Jr JW, Kazis L, Lee AF, Parchman ML, Zeber J, Pederson T, Montgomery KA, Hitchcock Noël P (2009). Multimorbidity clusters: clustering binary data from multimorbidity clusters: clustering binary data from a large administrative medical database. Appl Multivar Res.

[51] Brin S, Motwani R, Silverstein C (1997). Beyond market baskets: generalizing association rules to correlations. Proceedings of the 1997 ACM SIGMOD International Conference on Management of data.

[52] Moni MA, Liò P (2014). comoR: a software for disease comorbidity risk assessment. J Clin Bioinforma.

[53] Dunning AJ, Kensler J, Coudeville L, Bailleux F (2015). Some extensions in continuous models for immunological correlates of protection. BMC Med Res Methodol.

[54] Aguado A, Moratalla-Navarro F, López-Simarro F, Moreno V (2020). MorbiNet: multimorbidity networks in adult general population. Analysis of type 2 diabetes mellitus comorbidity. Sci Rep.

[55] Klimek P, Kautzky-Willer A, Chmiel A, Schiller-Frühwirth I, Thurner S (2015). Quantification of diabetes comorbidity risks across life using nation-wide big claims data. PLoS Comput Biol.

[56] HuDiNe Search.

[57] Bastian M, Heymann S, Jacomy M (2009). Gephi: an open source software for exploring and manipulating networks. Proceedings of the International AAAI Conference on Web and Social Media.

[58] Rózemberczki B, Davies R, Sarkar R, Sutton C (2019). GEMSEC: graph embedding with self clustering. Proceedings of the 2019 IEEE/ACM International Conference on Advances in Social Networks Analysis and Mining.

[59] Zhao Q, Fränti P (2014). WB-index: a sum-of-squares based index for cluster validity. Data Knowl Eng.

[60] Al- Zoubi MB, al Rawi M (2008). An efficient approach for computing silhouette coefficients. J Comput Sci.

[61] Corrigan P (2004). How stigma interferes with mental health care. Am Psychol.

[62] (2020). Findicator - Mortality from ischaemic heart disease. Statistics Finland.

[63] Karolaakso T, Autio R, Näppilä T, Nurmela K, Pirkola S (2020). Socioeconomic factors in disability retirement due to mental disorders in Finland. Eur J Public Health.

[64] (2021). Official Statistics of Finland (OSF): Births. Statistics Finland.

[65] Waters H, Graf M (2018). The costs of chronic disease in the U.S. The Milken Institute.

[66] Busija L, Lim K, Szoeke C, Sanders KM, McCabe MP (2019). Do replicable profiles of multimorbidity exist? Systematic review and synthesis. Eur J Epidemiol.

