# Supplementary material for: Clustering Diagnoses From 58 Million Patient Visits in Finland Between 2015 and 2018
Source: JMIR Med Inform. 2022 May 4;10(5):e35422. doi: 10.2196/35422 (PMC9118010; doi:10.2196/35422)
Supplement: Multimedia Appendix 1 [file medinform_v10i5e35422_app1.docx]

**Appendix I: ICD-10 blocks**

| **ICD-10 block** | **Description** |
| --- | --- |
| A00-A09 | Intestinal infectious diseases |
| A15-A19 | Tuberculosis |
| A20-A28 | Certain zoonotic bacterial diseases |
| A30-A49 | Other bacterial diseases |
| A50-A64 | Infections with a predominantly sexual mode of transmission |
| A65-A69 | Other spirochaetal diseases |
| A70-A74 | Other diseases caused by chlamydiae |
| A75-A79 | Rickettsioses |
| A80-A89 | Viral infections of the central nervous system |
| A90-A99 | Arthropod-borne viral fevers and viral haemorrhagic fevers |
| B00-B09 | Viral infections characterized by skin and mucous membrane lesions |
| B15-B19 | Viral hepatitis |
| B20-B24 | Human immunodeficiency virus [HIV] disease |
| B25-B34 | Other viral diseases |
| B35-B49 | Mycoses |
| B50-B64 | Protozoal diseases |
| B65-B83 | Helminthiases |
| B85-B89 | Pediculosis, acariasis and other infestations |
| B90-B94 | Sequelae of infectious and parasitic diseases |
| B95-B98 | Bacterial, viral and other infectious agents |
| B99-B99 | Other infectious diseases |
| C00-C14 | Malignant neoplasms of lip, oral cavity and pharynx |
| C15-C26 | Malignant neoplasms of digestive organs |
| C30-C39 | Malignant neoplasms of respiratory and intrathoracic organs |
| C40-C41 | Malignant neoplasms of bone and articular cartilage |
| C43-C44 | Melanoma and other malignant neoplasms of skin |
| C45-C49 | Malignant neoplasms of mesothelial and soft tissue |
| C50-C50 | Malignant neoplasm of breast |
| C51-C58 | Malignant neoplasms of female genital organs |
| C60-C63 | Malignant neoplasms of male genital organs |
| C64-C68 | Malignant neoplasms of urinary tract |
| C69-C72 | Malignant neoplasms of eye, brain and other parts of central nervous system |
| C73-C75 | Malignant neoplasms of thyroid and other endocrine glands |
| C76-C80 | Malignant neoplasms of ill-defined, secondary and unspecified sites |
| C81-C96 | Malignant neoplasms, stated or presumed to be primary, of lymphoid, haematopoietic and related tissue |
| C97-C97 | Malignant neoplasms of independent (primary) multiple sites |
| D00-D09 | In situ neoplasms |
| D10-D36 | Benign neoplasms |
| D37-D48 | Neoplasms of uncertain or unknown behaviour |
| D50-D53 | Nutritional anaemias |
| D55-D59 | Haemolytic anaemias |
| D60-D64 | Aplastic and other anaemias |
| D65-D69 | Coagulation defects, purpura and other haemorrhagic conditions |
| D70-D77 | Other diseases of blood and blood-forming organs |
| D80-D89 | Certain disorders involving the immune mechanism |
| E00-E07 | Disorders of thyroid gland |
| E10-E14 | Diabetes mellitus |
| E15-E16 | Other disorders of glucose regulation and pancreatic internal secretion |
| E20-E35 | Disorders of other endocrine glands |
| E40-E46 | Malnutrition |
| E50-E64 | Other nutritional deficiencies |
| E65-E68 | Obesity and other hyperalimentation |
| E70-E90 | Metabolic disorders |
| F00-F09 | Organic, including symptomatic, mental disorders |
| F10-F19 | Mental and behavioural disorders due to psychoactive substance use |
| F20-F29 | Schizophrenia, schizotypal and delusional disorders |
| F30-F39 | Mood [affective] disorders |
| F40-F48 | Neurotic, stress-related and somatoform disorders |
| F50-F59 | Behavioural syndromes associated with physiological disturbances and physical factors |
| F60-F69 | Disorders of adult personality and behaviour |
| F70-F79 | Mental retardation |
| F80-F89 | Disorders of psychological development |
| F90-F98 | Behavioural and emotional disorders with onset usually occurring in childhood and adolescence |
| F99-F99 | Unspecified mental disorder |
| G00-G09 | Inflammatory diseases of the central nervous system |
| G10-G14 | Systemic atrophies primarily affecting the central nervous system |
| G20-G26 | Extrapyramidal and movement disorders |
| G30-G32 | Other degenerative diseases of the nervous system |
| G35-G37 | Demyelinating diseases of the central nervous system |
| G40-G47 | Episodic and paroxysmal disorders |
| G50-G59 | Nerve, nerve root and plexus disorders |
| G60-G64 | Polyneuropathies and other disorders of the peripheral nervous system |
| G70-G73 | Diseases of myoneural junction and muscle |
| G80-G83 | Cerebral palsy and other paralytic syndromes |
| G90-G99 | Other disorders of the nervous system |
| H00-H06 | Disorders of eyelid, lacrimal system and orbit |
| H10-H13 | Disorders of conjunctiva |
| H15-H22 | Disorders of sclera, cornea, iris and ciliary body |
| H25-H28 | Disorders of lens |
| H30-H36 | Disorders of choroid and retina |
| H40-H42 | Glaucoma |
| H43-H45 | Disorders of vitreous body and globe |
| H46-H48 | Disorders of optic nerve and visual pathways |
| H49-H52 | Disorders of ocular muscles, binocular movement, accommodation and refraction |
| H53-H54 | Visual disturbances and blindness |
| H55-H59 | Other disorders of eye and adnexa |
| H60-H62 | Diseases of external ear |
| H65-H75 | Diseases of middle ear and mastoid |
| H80-H83 | Diseases of inner ear |
| H90-H95 | Other disorders of ear |
| I00-I02 | Acute rheumatic fever |
| I05-I09 | Chronic rheumatic heart diseases |
| I10-I15 | Hypertensive diseases |
| I20-I25 | Ischaemic heart diseases |
| I26-I28 | Pulmonary heart disease and diseases of pulmonary circulation |
| I30-I52 | Other forms of heart disease |
| I60-I69 | Cerebrovascular diseases |
| I70-I79 | Diseases of arteries, arterioles and capillaries |
| I80-I89 | Diseases of veins, lymphatic vessels and lymph nodes, not elsewhere classified |
| I95-I99 | Other and unspecified disorders of the circulatory system |
| J00-J06 | Acute upper respiratory infections |
| J09-J18 | Influenza and pneumonia |
| J20-J22 | Other acute lower respiratory infections |
| J30-J39 | Other diseases of upper respiratory tract |
| J40-J47 | Chronic lower respiratory diseases |
| J60-J70 | Lung diseases due to external agents |
| J80-J84 | Other respiratory diseases principally affecting the interstitium |
| J85-J86 | Suppurative and necrotic conditions of lower respiratory tract |
| J90-J94 | Other diseases of pleura |
| J95-J99 | Other diseases of the respiratory system |
| K00-K14 | Diseases of oral cavity, salivary glands and jaws |
| K20-K31 | Diseases of oesophagus, stomach and duodenum |
| K35-K38 | Diseases of appendix |
| K40-K46 | Hernia |
| K50-K52 | Noninfective enteritis and colitis |
| K55-K64 | Other diseases of intestines |
| K65-K67 | Diseases of peritoneum |
| K70-K77 | Diseases of liver |
| K80-K87 | Disorders of gallbladder, biliary tract and pancreas |
| K90-K93 | Other diseases of the digestive system |
| L00-L08 | Infections of the skin and subcutaneous tissue |
| L10-L14 | Bullous disorders |
| L20-L30 | Dermatitis and eczema |
| L40-L45 | Papulosquamous disorders |
| L50-L54 | Urticaria and erythema |
| L55-L59 | Radiation-related disorders of the skin and subcutaneous tissue |
| L60-L75 | Disorders of skin appendages |
| L80-L99 | Other disorders of the skin and subcutaneous tissue |
| M00-M03 | Infectious arthropathies |
| M05-M14 | Inflammatory polyarthropathies |
| M15-M19 | Arthrosis |
| M20-M25 | Other joint disorders |
| M30-M36 | Systemic connective tissue disorders |
| M40-M43 | Deforming dorsopathies |
| M45-M49 | Spondylopathies |
| M50-M54 | Other dorsopathies |
| M60-M63 | Disorders of muscles |
| M65-M68 | Disorders of synovium and tendon |
| M70-M79 | Other soft tissue disorders |
| M80-M85 | Disorders of bone density and structure |
| M86-M90 | Other osteopathies |
| M91-M94 | Chondropathies |
| M95-M99 | Other disorders of the musculoskeletal system and connective tissue |
| N00-N08 | Glomerular diseases |
| N10-N16 | Renal tubulo-interstitial diseases |
| N17-N19 | Renal failure |
| N20-N23 | Urolithiasis |
| N25-N29 | Other disorders of kidney and ureter |
| N30-N39 | Other diseases of urinary system |
| N40-N51 | Diseases of male genital organs |
| N60-N64 | Disorders of breast |
| N70-N77 | Inflammatory diseases of female pelvic organs |
| N80-N98 | Noninflammatory disorders of female genital tract |
| N99-N99 | Other disorders of the genitourinary system |
| O00-O08 | Pregnancy with abortive outcome |
| O10-O16 | Oedema, proteinuria and hypertensive disorders in pregnancy, childbirth and the puerperium |
| O20-O29 | Other maternal disorders predominantly related to pregnancy |
| O30-O48 | Maternal care related to the fetus and amniotic cavity and possible delivery problems |
| O60-O75 | Complications of labour and delivery |
| O80-O84 | Delivery |
| O85-O92 | Complications predominantly related to the puerperium |
| O94-O99 | Other obstetric conditions, not elsewhere classified |
| P00-P04 | Fetus and newborn affected by maternal factors and by complications of pregnancy, labour and delivery |
| P05-P08 | Disorders related to length of gestation and fetal growth |
| P10-P15 | Birth trauma |
| P20-P29 | Respiratory and cardiovascular disorders specific to the perinatal period |
| P35-P39 | Infections specific to the perinatal period |
| P50-P61 | Haemorrhagic and haematological disorders of fetus and newborn |
| P70-P74 | Transitory endocrine and metabolic disorders specific to fetus and newborn |
| P80-P83 | Conditions involving the integument and temperature regulation of fetus and newborn |
| P90-P96 | Other disorders originating in the perinatal period |
| Q00-Q07 | Congenital malformations of the nervous system |
| Q10-Q18 | Congenital malformations of eye, ear, face and neck |
| Q20-Q28 | Congenital malformations of the circulatory system |
| Q30-Q34 | Congenital malformations of the respiratory system |
| Q35-Q37 | Cleft lip and cleft palate |
| Q38-Q45 | Other congenital malformations of the digestive system |
| Q50-Q56 | Congenital malformations of genital organs |
| Q60-Q64 | Congenital malformations of the urinary system |
| Q65-Q79 | Congenital malformations and deformations of the musculoskeletal system |
| Q80-Q89 | Other congenital malformations |
| Q90-Q99 | Chromosomal abnormalities, not elsewhere classified |
| S00-S09 | Injuries to the head |
| S10-S19 | Injuries to the neck |
| S20-S29 | Injuries to the thorax |
| S30-S39 | Injuries to the abdomen, lower back, lumbar spine and pelvis |
| S40-S49 | Injuries to the shoulder and upper arm |
| S50-S59 | Injuries to the elbow and forearm |
| S60-S69 | Injuries to the wrist and hand |
| S70-S79 | Injuries to the hip and thigh |
| S80-S89 | Injuries to the knee and lower leg |
| S90-S99 | Injuries to the ankle and foot |
| T00-T07 | Injuries involving multiple body regions |
| T08-T14 | Injuries to unspecified part of trunk, limb or body region |
| T15-T19 | Effects of foreign body entering through natural orifice |
| T20-T25 | Burns and corrosions of external body surface, specified by site |
| T26-T28 | Burns and corrosions confined to eye and internal organs |
| T29-T32 | Burns and corrosions of multiple and unspecified body regions |
| T33-T35 | Frostbite |
| T36-T50 | Poisoning by drugs, medicaments and biological substances |
| T51-T65 | Toxic effects of substances chiefly nonmedicinal as to source |
| T66-T78 | Other and unspecified effects of external causes |
| T79-T79 | Certain early complications of trauma |
| T80-T88 | Complications of surgical and medical care, not elsewhere classified |
| T90-T98 | Sequelae of injuries, of poisoning and of other consequences of external causes |
